# Distributed representations of temporally accumulated reward prediction errors in the mouse cortex

**DOI:** 10.1126/sciadv.adi4782

**Published:** 2025-01-22

**Authors:** Hiroshi Makino, Ahmad Suhaimi

**Affiliations:** ^1^ Lee Kong Chian School of Medicine, Nanyang Technological University, 11 Mandalay Road, Singapore 308232, Singapore.; ^2^ Department of Physiology, Keio University School of Medicine, Tokyo 160-8582, Japan.

## Abstract

Reward prediction errors (RPEs) quantify the difference between expected and actual rewards, serving to refine future actions. Although reinforcement learning (RL) provides ample theoretical evidence suggesting that the long-term accumulation of these error signals improves learning efficiency, it remains unclear whether the brain uses similar mechanisms. To explore this, we constructed RL-based theoretical models and used multiregional two-photon calcium imaging in the mouse dorsal cortex. We identified a population of neurons whose activity was modulated by varying degrees of RPE accumulation. Consequently, RPE-encoding neurons were sequentially activated within each trial, forming a distributed assembly. RPE representations in mice aligned with theoretical predictions of RL, emerging during learning and being subject to manipulations of the reward function. Interareal comparisons revealed a region-specific code, with higher-order cortical regions exhibiting long-term encoding of RPE accumulation. These results present an additional layer of complexity in cortical RPE computation, potentially augmenting learning efficiency in animals.

## Teaser

Region-specific cortical representations of temporally accumulated reward prediction errors were identified.

## INTRODUCTION

Behavior optimization involves evaluating the outcomes of executed actions and making error-based adjustments to subsequent actions (*1*). Reinforcement learning (RL) offers a theoretical framework for studying the improvement of control policies through a trial-and-error process (*2*–*4*). For instance, the temporal difference (TD) error in computational RL has provided better interpretations of the phasic activity of dopamine neurons in response to unpredicted rewards, known as reward prediction errors (RPEs) (*5*–*16*).

In computational RL, the TD error typically refers to a single-step TD error, representing the difference between an agent’s current value estimate and the updated value estimate based on the next state and reward (*17*). However, to better control the bias-variance trade-off, the TD error can be extended to a multistep (n-step) TD error (*17*), which can be expressed as an accumulation of single-step TD errors. A mixed code of various levels of TD error accumulation has been shown to improve learning efficiency in artificial agents (*18*).

In neuroscience, increasing evidence suggests that signals related to RPEs, whether positive or negative, are also present in the cortex (*19*–*21*). However, despite extensive studies on dopamine neurons and associated subcortical structures (*22*, *23*), cortical representations of RPEs remain underexplored. In particular, it is poorly understood whether representations of temporally accumulated RPEs are present in the cortex.

In this study, we constructed deep RL-based theoretical models to investigate the nature of error signals in motor control and whether RPE accumulation is encoded in the mouse cortex. We examined neural representations of RPEs across multiple regions of the mouse cortex while the mice remotely manipulated an object. We found that individual cortical neurons responded to varying degrees of RPE accumulation, forming a distributed sequential assembly within each trial. Consistent with the idea that neurons represent RPEs, the error-evoked activity was subject to modifications in reward functions, with larger discrepancies between expected and actual rewards evoking activity in more neurons. Notably, higher-order cortical regions, such as the retrosplenial cortex (RSC) and secondary motor cortex (M2), displayed coding for accumulated RPEs over an extended period. These results reveal a neural code for distributed cortical processing of RPE accumulation.

## RESULTS

### Error accumulation in an object manipulation task

**Fig. 1.**
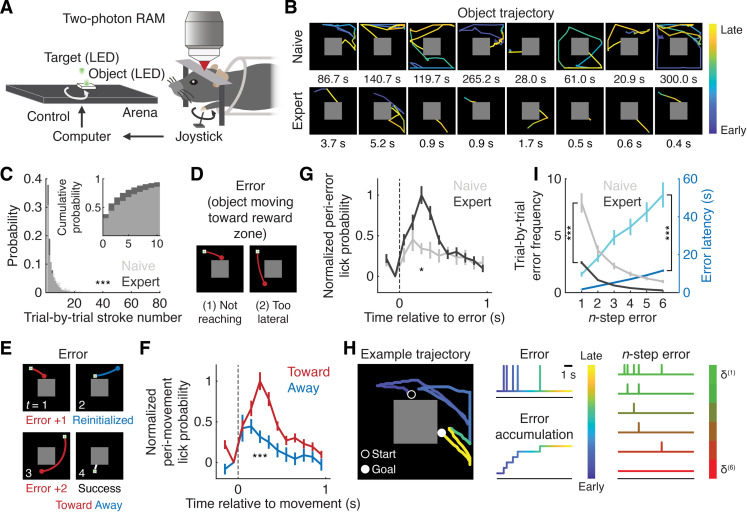
Error accumulation in the object manipulation task. (**A**) Schematic of the object manipulation task. Mice use a joystick to move an object to the reward zone (target) located in the center of the arena. RAM denotes random access mesoscope. (**B**) Example object trajectories and their durations in eight consecutive trials for naive and expert mice. (**C**) Histogram and cumulative probability (inset) of trial-by-trial stroke number (***P < 0.001, naive: *n* = 761 trials from five mice; expert: *n* = 4500 trials from nine mice, one-tailed Kolmogorov-Smirnov test). (**D**) Schematic of the two types of erroneous object movement. (**E**) Schematic of error accumulation over time (t). Object movements away from the reward zone were considered reinitialization. (**F**) Normalized peri–movement lick probability (***P < 0.001, *n* = 75 sessions from nine mice, one-tailed Wilcoxon signed-rank test, means ± SEM). (**G**) Normalized peri–error lick probability (*P < 0.05, naive: *n* = 19 sessions from five mice; expert: *n* = 75 sessions from nine mice, one-tailed Wilcoxon rank sum test, means ± SEM). (**H**) (Left) Object trajectory, single-step error, and error accumulation in an example trial. (Right) Resulting n-step error onsets in the example trial. (**I**) Trial-by-trial error frequency (***P < 0.001 for all n steps, naive: *n* = 19 sessions from five mice; expert: *n* = 75 sessions from nine mice for one to six steps, one-tailed bootstrap with Bonferroni correction, means ± SEM) and error latency (***P < 0.001 for all n steps, naive: *n* = 19 sessions from five mice for one to six steps; expert: *n* = 75 sessions for one to five steps and 73 sessions for six steps from nine mice, one-tailed bootstrap with Bonferroni correction, means ± SEM) as a function of n steps.

We trained head-restrained mice to perform an object manipulation task, where they used a joystick to guide a light-emitting diode (LED)–attached object toward a reward zone (4 by 4 cm^2^) located at the center of an arena (10 by 10 cm^2^; Fig. 1A) (*24*, *25*). At the beginning of each trial, the object was randomly placed outside the reward zone. Mice received an 8-μl water reward when they successfully guided the object into the reward zone and the object became stationary. Trials ended if this was not achieved within 5 min. Each session consisted of up to 60 trials.

Mice progressively improved in the task, as indicated by increased correct rates (*P* < 0.001, naive: 61.1 ± 7.6%, *n* = 19 sessions from five mice; expert: 99.9 ± 0.05%, *n* = 75 sessions from nine mice, one-tailed bootstrap, means ± SEM) and a decreased number of “strokes” per trial, defined as object movements exceeding a predetermined speed threshold (*P* < 0.001, naive: 5.6 ± 0.2, *n* = 761 trials from five mice; expert: 3.7 ± 0.1, *n* = 4500 trials from nine mice, one-tailed Kolmogorov-Smirnov test, means ± SEM; Fig. 1, B and C).

Expert mice typically completed each trial with a few strokes but often made erroneous movements due to either (i) insufficient object movement to reach the reward zone or (ii) misaimed lateral movement (Fig. 1D). Object movements away from the reward zone were considered reinitialization of the object’s position because the anticipatory lick probability, a proxy for reward expectation, was lower after these movements (“away”) than after the erroneous movements (“toward”) (*P* < 0.001, *n* = 75 sessions from nine mice, one-tailed Wilcoxon signed-rank test; Fig. 1, E and F). Thus, we considered the behavioral task to correspond to a multiple center-in task.

The likelihood of post–error licking increased over the course of learning (*P* < 0.05, naive: *n* = 19 sessions from five mice; expert: *n* = 75 sessions from nine mice, one-tailed Wilcoxon rank sum test; Fig. 1G), suggesting augmented reward anticipation in expert mice. These results suggest that, through learning, mice developed a reward expectation when they moved the object toward, but not away from, the reward zone. Because the mice failed to obtain the expected reward after each erroneous movement, we considered these instances as corresponding to negative RPEs.

Errors could occur multiple times (n times) within each trial, and we considered the accumulation of these errors over time as an n-step error (Fig. 1H). For example, if the error occurred five times within a trial, then it was identified as a five-step error. Expert mice consistently demonstrated lower trial-by-trial error frequency and error latency, defined as the time between two consecutive errors for each n-step, across all n steps compared to naive mice (frequency: *P* < 0.001 for all n steps, naive: *n* = 19 sessions from five mice; expert: *n* = 75 sessions from nine mice for one to six steps, one-tailed bootstrap with Bonferroni correction; latency: *P* < 0.001 for all *n*-steps, naive: *n* = 19 sessions from five mice; expert: *n* = 75 sessions for one to five steps and 73 sessions from nine mice for six steps, one-tailed bootstrap with Bonferroni correction; Fig. 1I). Trial-by-trial error frequency in naive and expert mice remained unchanged within each session (naive: *P* > 0.05 for all *n*-steps, *n* = 19, 18, 13, and 8 sessions for the first, second, third, and fourth quarter from five mice, respectively; expert: *P* > 0.05 for all *n*-steps, *n* = 75 sessions for all quarters from nine mice, Kruskal-Wallis test with false discovery rate; fig. S1A). Furthermore, post–error licking in expert mice remained stable across n-step errors (*P* = 0.30, *n* = 75 sessions for one to five steps and 71 sessions for six steps from nine mice, Kruskal-Wallis test; fig. S1A) and within each session (*P* = 0.60, *n* = 75 sessions for all quarters from nine mice, Kruskal-Wallis test; fig. S1A).

### Learning in artificial agents through RPE accumulation

**Fig. 2.**
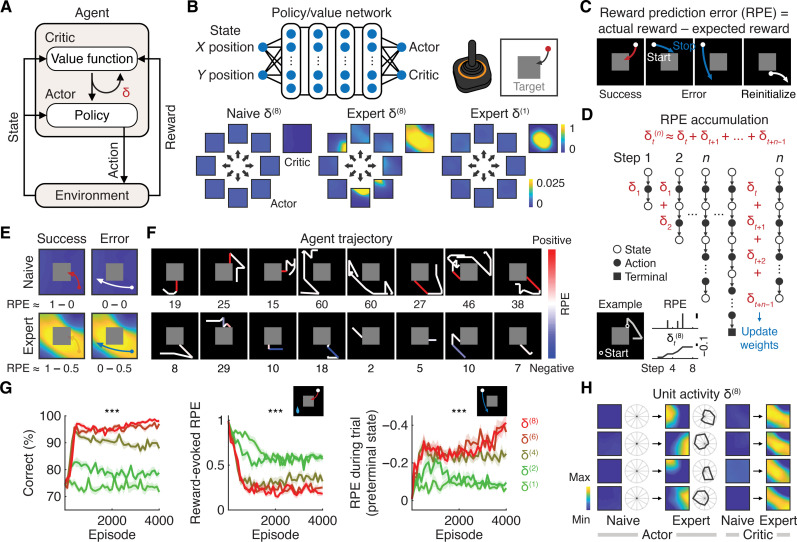
Learning in artificial agents with RPE accumulation. (**A**) Schematic of the actor-critic architecture. The agent interacts with the environment through actions. δ represents the RPE. (**B**) (Top) Schematic of the artificial neural network of the agent and task, with the gray square indicating the reward zone. (Bottom) Examples of actor and critic output of the neural network across different conditions. (**C**) Schematic of RPEs as defined in RL. Object movements away from the reward zone were considered reinitialization. (**D**) An n-step RPE ($\delta_{t}^{\left(n\right)}$) can be interpreted as an accumulation of single-step RPEs. Inset illustrates example RPE accumulation over time. (**E**) Schematic of RPEs with successful and erroneous movements in naive and expert agents. (**F**) Example trajectories and step numbers in eight trials for naive and expert agents [$\delta^{\left(8\right)}$]. Note that most steps in the agent corresponded to no movement and that reward-evoked RPEs (red) became less positive, whereas RPEs before reaching the reward zone (blue) became more negative over learning. (**G**) (Left) Learning curves across different n (***P < 0.001, *n* = 10 agents, Kruskal-Wallis test, means ± SEM). (Middle) Learning-related changes in reward-evoked RPE across different n (***P < 0.001, *n* = 10 agents, Kruskal-Wallis test, means ± SEM). (Right) Learning-related changes in RPE before reaching the reward zone across different n (***P < 0.001, *n* = 10 agents, Kruskal-Wallis test, means ± SEM). (**H**) Examples of the agent’s unit representation in space and direction of actor and critic over learning with $\delta^{\left(8\right)}$.

To theoretically establish how error accumulation influences learning, we constructed RL models using a variant of the proximal policy optimization (PPO) algorithm (Fig. 2, A and B). PPO is a model-free deep RL algorithm that combines policy gradient and value-based methods using an advantage function (*26*). The advantage function determines the relative value of an action in each state compared to the expected value of all possible actions in that state. In deep RL, n-step TD errors (or equivalently, advantage estimates) can be expressed as an accumulation of single-step TD errors. Combining multiple n-step TD errors with different horizons offers a more stable and effective advantage estimation for policy improvement (*18*).

As in the case of the mice, we designed an environment for agents where a single action could suffice to reach the terminal state. We considered single-step negative RPEs in cases where agents either (i) failed to move far enough to reach the reward zone or (ii) aimed too laterally (Fig. 2C). Because we aimed to model RPE accumulation as observed in mice, we did not use the canonical TD error, which is computed by the difference between value estimates of consecutive states. Instead, we implemented RPE, defined as the difference between the actual reward and the expected reward (i.e., the estimated value of each state), in the PPO algorithm. The weights of the actor-critic neural network were updated with RPE accumulation over n steps (Fig. 2D and fig. S2A). We also considered positive RPEs when agents unexpectedly obtained a reward by reaching the reward zone (Fig. 2E). Agents optimized their behavioral policies by repeating actions that led to positive RPEs while avoiding those that resulted in negative RPEs.

Learning improved with the accumulation of RPEs over time within each trial (*P* < 0.001, *n* = 10 agents, Kruskal-Wallis test; Fig. 2, F and G, and fig. S3A). Over the course of learning, reward-evoked positive RPEs decreased due to enhanced reward expectation (eight steps: *P* < 0.001 between naive and expert, *n* = 10 agents, one-tailed paired bootstrap; Fig. 2, F and G). In contrast, whereas erroneous movements before reaching the reward zone did not cause negative RPEs in naive agents, similar movements in expert agents generated negative RPEs (eight steps: *P* < 0.001 between naive and expert, *n* = 10 agents, one-tailed paired bootstrap; Fig. 2, F and G). These results were consistent across different hyperparameters, except when the critic loss coefficient or entropy coefficient in PPO was set to 0 (fig. S3B). Furthermore, each unit in the neural network showed actor or critic representations after learning (Fig. 2H and fig. S2, B and C). Together, our RL analysis highlights the importance of RPE accumulation in learning and establishes a theoretical foundation for RPE representations in mice.

### Emergence of cortical error representations over learning

**Fig. 3.**
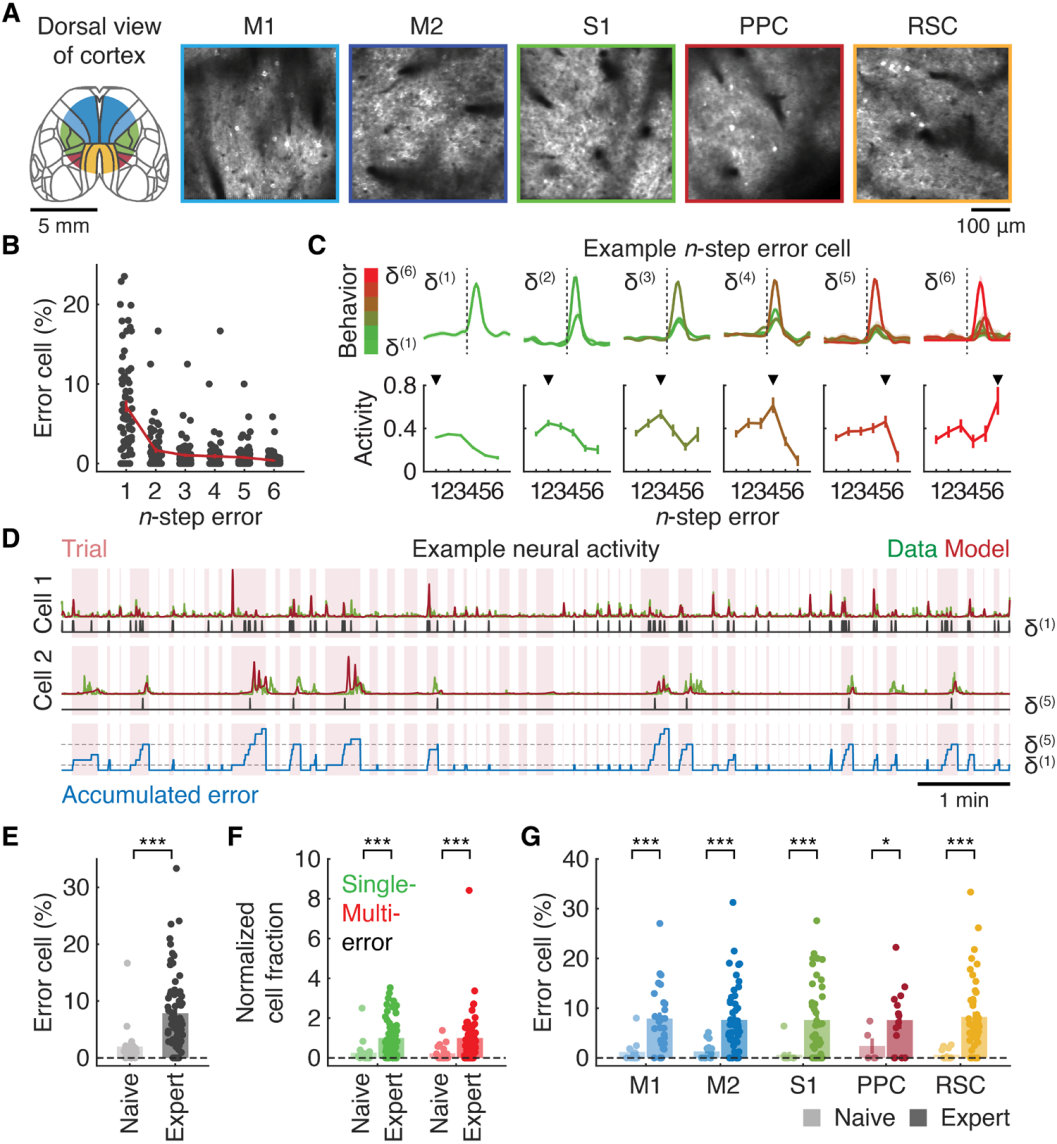
Emergence of cortical error representations over learning. (**A**) Example images of GCaMP6s in cortical regions. M1, primary motor cortex; M2, secondary motor cortex; S1, primary somatosensory cortex; PPC, posterior parietal cortex; RSC, retrosplenial cortex. (**B**) Fractions of error cells across n in expert mice (*n* = 70 sessions from nine mice). (**C**) (Top) Tuning specificity of example error neurons. (Bottom) Tuning curves of error neurons [$\delta^{\left(1\right)}$ to $\delta^{\left(5\right)}$: *n* = 1262, 224, 173, 127, 131, and 63 neurons; $\delta^{\left(6\right)}$: *n* = 1250, 221, 170, 127, 130, and 63 neurons from left to right from nine mice]. (**D**) Example session displaying *z*-scored activity derived from the deconvolved calcium signal of an entire session (data) or GLMs (model) of neurons encoding a single-step error (cell 1) or five-step error accumulation (cell 2). $\delta^{\left(1\right)}$ and $\delta^{\left(5\right)}$ describe the onsets of single-step and five-step errors, respectively. The blue line indicates trial-by-trial error accumulation based on the object trajectory, and the red box denotes the trial epoch. (**E**) Fractions of error neurons in naive and expert mice (***P < 0.001, naive: *n* = 19 sessions from five mice; expert: *n* = 70 sessions from nine mice, one-tailed bootstrap, means ± SEM). (**F**) Fractions of single-step and multistep error neurons normalized to expert fractions (***P < 0.001, naive: *n* = 19 sessions from five mice; expert: *n* = 70 sessions from nine mice, one-tailed bootstrap with Bonferroni correction, means ± SEM). (**G**) Fractions of error neurons in each cortical region (*P < 0.05; ***P < 0.001, naive: M1: 11, M2: 17, S1: 11, PPC: 5, and RSC: 13 sessions from five mice; expert: M1: 27, M2: 48, S1: 44, PPC: 14, and RSC: 48 sessions from nine mice, one-tailed bootstrap with Bonferroni correction, means ± SEM).

After theoretically establishing the importance of RPE accumulation, we next investigated its neural substrates by performing calcium imaging to probe the activity of excitatory neurons in transgenic mice (CaMKII-tTA × TRE-GCaMP6s) using a two-photon random access mesoscope (2p-RAM) (*27*). The imaging window included five cortical regions: the primary motor cortex (M1), M2, primary somatosensory cortex (S1), posterior parietal cortex (PPC), and RSC (Fig. 3A).

We constructed generalized linear models (GLMs) (*28*, *29*) for individual neurons using n-step errors, $\delta_{t}^{\left(n\right)}$, as predictors, where n (ranging from 1 to 6) denotes the number of single-step errors accumulated within each trial. We assumed that the value remained consistent for each erroneous movement as its moment-by-moment extraction was not feasible. Object velocity, joystick velocity, and passage of time were included as additional predictors in the GLM. Each model’s performance was quantitatively assessed by computing the pseudo-explained variance (E.V.) on data held out from the model fitting procedure (*30*). In total, 42.0 ± 1.8% (*n* = 72 sessions from nine mice, means ± SEM) of analyzed neurons in expert mice were significantly modulated by task variables and defined as task-related cells (M1: 4725 neurons; M2: 2935 neurons; S1: 4777 neurons; PPC: 520 neurons; and RSC: 2845 neurons; fig. S4, A and B). Task-related cells were further classified as error-related cells (17.0 ± 1.3% among analyzed neurons, *n* = 72 sessions from nine mice, means ± SEM) when removing error predictors from the GLM significantly deteriorated model performance.

To eliminate potential movement-related covariates, neurons were not considered error-related if the coefficients for object or joystick velocity predictors in the GLM were higher than those for error predictors. Furthermore, we fit separate GLMs by replacing the original error predictors (object moving “toward” the reward zone but failing to reach the target) with reinitialization predictors (object moving “away” from the reward zone). Neurons were considered to represent errors if the coefficients for error predictors were higher than those for control predictors. Thus, it is unlikely that the error neurons simply encoded a combination of movements and reward. Because of these strict criteria to avoid false positives, the resulting fraction of error neurons (Fig. 3B) is likely underestimated.

The error code in these neurons was highly specific, as demonstrated by their tuning curves (Fig. 3C). Because each neuron could encode multiple n-step errors, we further classified error neurons by assigning only one n per neuron, determined by computing the argmax of error coefficients across n in the GLM. By marginalizing activity related to nonerror task variables (object velocity, joystick velocity, and passage of time), we factorized the multiplexed representation and computed a model-derived response profile for n-step errors in each error neuron (Fig. 3D) (*31*).

Notably, when we examined the deconvolved calcium signals of error neurons in response to single-step errors, their temporal profiles and magnitudes were distinct from the calcium signals aligned with the offset of single-strokes in rewarded trials (fig. S5). This finding confirms that the responses of the error neurons are unlikely to be attributed to other covariates such as the movement termination, reward, or licking behavior.

Next, we investigated how these error representations emerged during learning. Similar to the artificial agents, if neural activity reflected negative RPEs, then it should be reduced in naive mice as they had not yet learned the state value (Fig. 2B). To explore this, we imaged neural activity in naive mice (task-related cells: 53.1 ± 4.2%, *n* = 19 sessions from five mice, means ± SEM) and built a GLM for each neuron.

GLM analysis revealed that the pseudo-E.V. of neurons in naive mice was lower than that of neurons in expert mice across cortical regions (*P* < 0.001 in all regions, two-tailed Kolmogorov-Smirnov test with Bonferroni correction; fig. S4A). The fraction of error neurons in naive mice was significantly lower, regardless of whether they were encoding single-step or multistep errors (*P* < 0.001, naive: *n* = 19 sessions from five mice; expert: 70 sessions from nine mice, one-tailed bootstrap with Bonferroni correction; Fig. 3, E and F). A region-by-region analysis revealed a global reduction in the fraction of error neurons across all five cortical regions (*P* < 0.05 for PPC, *P* < 0.001 for other regions, expert: M1: 27; M2: 48; S1: 44; PPC: 14; and RSC: 48 sessions from nine mice; naive: M1: 11; M2: 17; S1: 11; PPC: 5; and RSC: 13 sessions from five mice, one-tailed bootstrap with Bonferroni correction; Fig. 3G). Because naive mice exhibited a higher error frequency (Fig. 1I), the error representations observed in neurons of expert mice are unlikely to be attributed to the erroneous object movements themselves. Instead, these findings suggest that cortical neurons encoded the accumulation of RPEs, presumably due to increased poststroke reward anticipation over learning (Fig. 1G).

Furthermore, similar to artificial agents showing reduced reward-evoked RPEs due to increased reward expectation (Fig. 2, F and G), reward-evoked activity in cortical neurons generally decreased over the course of learning (M1: *P* < 0.05, *n* = 1077, 7619 neurons; M2: *P* < 0.001, *n* = 1761, 6415 neurons; S1: *P* < 0.001, *n* = 797, 7785 neurons; PPC: *P* < 0.001, *n* = 207, 1081 neurons; and RSC: *P* < 0.001, *n* = 1098, 8776 neurons from five naive and nine expert mice, one-tailed bootstrap with false discovery rate; fig. S6). These results suggest that neural responses in mice were strongly influenced by reward expectation.

### Sequential activity of distributed cortical neurons for error representations

**Fig. 4.**
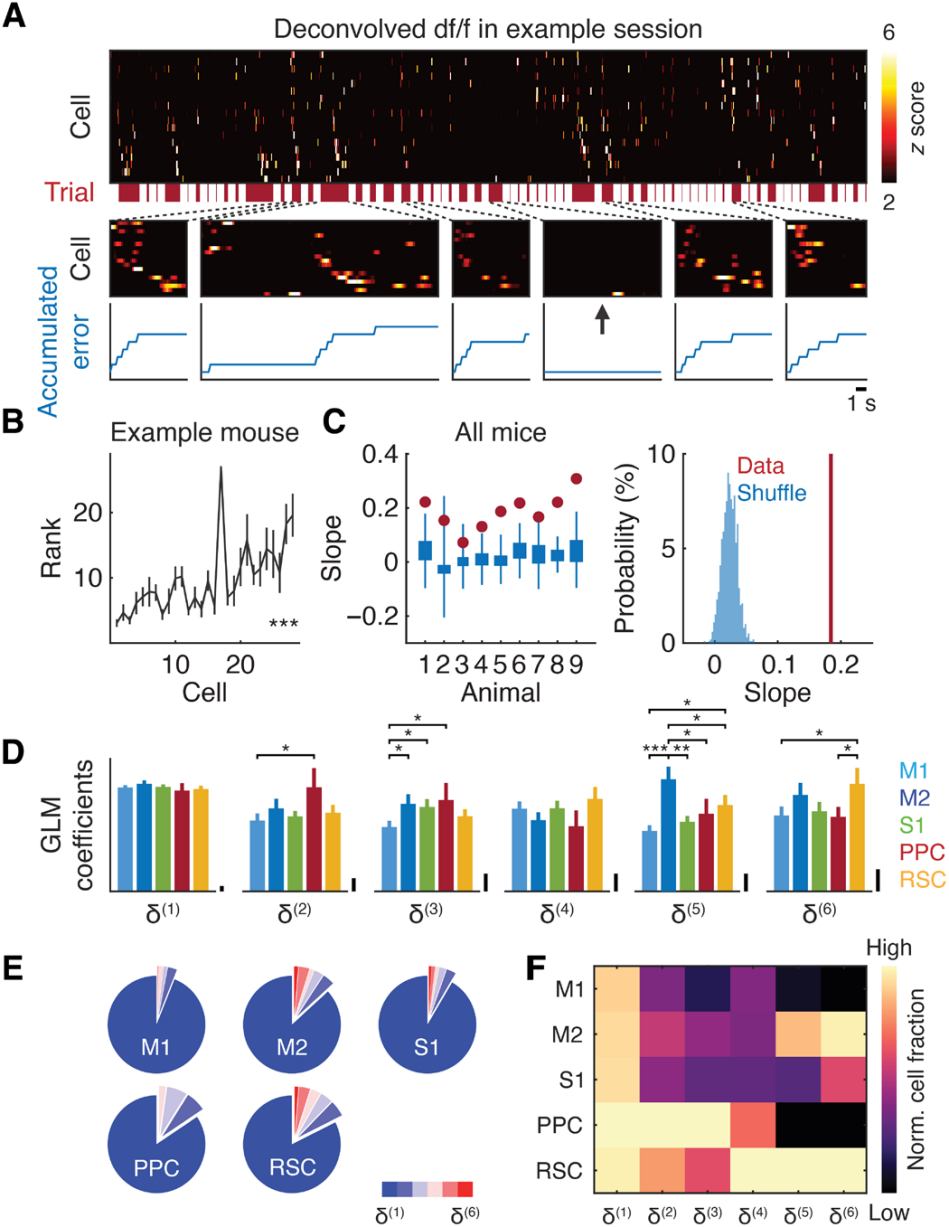
Sequential activity and area-specific code for error representations. (**A**) Example session displaying trial-by-trial sequential activity of cortical neurons. (Top) *z*-scored deconvolved df/f (change in fluorescence) of error neurons. Trial epochs are indicated by the red boxes. (Middle) Example trials. The arrow highlights a long trial without errors, coinciding with the absence of neural activity. (Bottom) Trial-by-trial error accumulation derived from the object trajectory. (**B**) Mean rank of the activity onset of neurons across trials as a function of the sorting index from the example session in (A) (***P < 0.001, one-tailed permutation). (**C**) (Left) Sequential activity derived from deconvolved df/f is not explained by chance in individual mice (mouse 1 and 4 to 9: *P* < 0.001; mouse 2: *P* < 0.05; and mouse 3: *P* = 0.06, one-tailed permutation). The whisker edges represent maximum and minimum values, and the box edges represent 75 and 25% of 1000 shuffled mean slopes. (Right) Mean slope computed from all mice and mean slope derived from shuffled data of all mice (*P* < 0.001, *n* = 9 mice, one-tailed permutation). (**D**) GLM coefficients for n-step errors in neurons without argmax sorting in each cortical region (*P < 0.05; **P < 0.01; ***P < 0.001, $\delta^{\left(1\right)}$: *n* = 340, 244, 390, 51, and 249 neurons; $\delta^{\left(2\right)}$: *n* = 82, 84, 124, 20, and 102 neurons; $\delta^{\left(3\right)}$: *n* = 95, 91, 109, 18, and 97 neurons; $\delta^{\left(4\right)}$: *n* = 103, 76, 98, 12, and 66 neurons; $\delta^{\left(5\right)}$: *n* = 98, 81, 130, 17, and 81 neurons; and $\delta^{\left(6\right)}$: *n* = 79, 52, 84, 14, and 60 neurons from left to right from nine mice, one-tailed bootstrap, means ± SEM). Scale bars, 0.01. (**E**) Distribution of neurons encoding different n-step errors in each cortical region. (**F**) Fractions of error neurons normalized to the maximum across regions in each n-step error.

We hypothesized that, if each neuron responded to a distinct level of error accumulation, a population of error neurons should exhibit sequential activation. Using raw deconvolved calcium signals, we confirmed this on a trial-by-trial basis (Fig. 4A and fig. S7A). Activity was nearly absent when error accumulation was minimal, even in long trials (Fig. 4A and fig. S7A), suggesting that the neurons’ activation was not simply due to the passage of time.

To quantify the sequential activity of neurons, we sorted them based on their activity onset order in half of the trials in each session and applied the sorting index to the other half of the trials. We then computed a slope between the sorting index and the trial-averaged order of these neurons, where a slope of 1 indicated consistent sequential activity across trials. This analysis revealed that neural population activity was temporally organized, a pattern not explained by chance, as shuffling the activity did not produce such a positive relationship in eight of nine mice (*P* < 0.05 for one mouse, *P* < 0.001 for seven mice, and *P* = 0.06 for one mouse, one-tailed permutation with false discovery rate; Fig. 4, B and C) or in the population of mice (*P* < 0.001, *n* = 9 mice, one-tailed permutation; Fig. 4C). The same trend was also observed in GLM-derived activity in individual mice (*P* < 0.001 in all nine mice, one-tailed permutation with Bonferroni correction; fig. S7, A to C) and in the population of mice (*P* < 0.001, *n* = 9 mice, one-tailed permutation; fig. S7C). Furthermore, we confirmed that the neural activity of error neurons was higher in long trials containing errors than in those without errors (*P* < 0.001, *n* = 1333 cells from nine mice, one-tailed bootstrap; fig. S7D). These results reveal a cortex-wide sequential assembly encoding error accumulation, independent of the passage of time.

Next, we examined whether each cortical region represented different levels of error accumulation. The GLM coefficients for the n-step errors in neurons displayed distinct distributions across regions. For instance, M2 and RSC neurons had large coefficients for long-term error accumulation, whereas PPC neurons exhibited large coefficients for intermediate horizons of error accumulation (one-tailed bootstrap; Fig. 4D). Although single-step error representations were prevalent in each region, a similar trend was observed when the fractions of neurons encoding the n-step errors were computed (Fig. 4, E and F). Our results, therefore, identified area-specific representations of error accumulation and highlighted the unique code for long-term error accumulation in higher-order cortical regions.

### Reward dependency of error representations

**Fig. 5.**
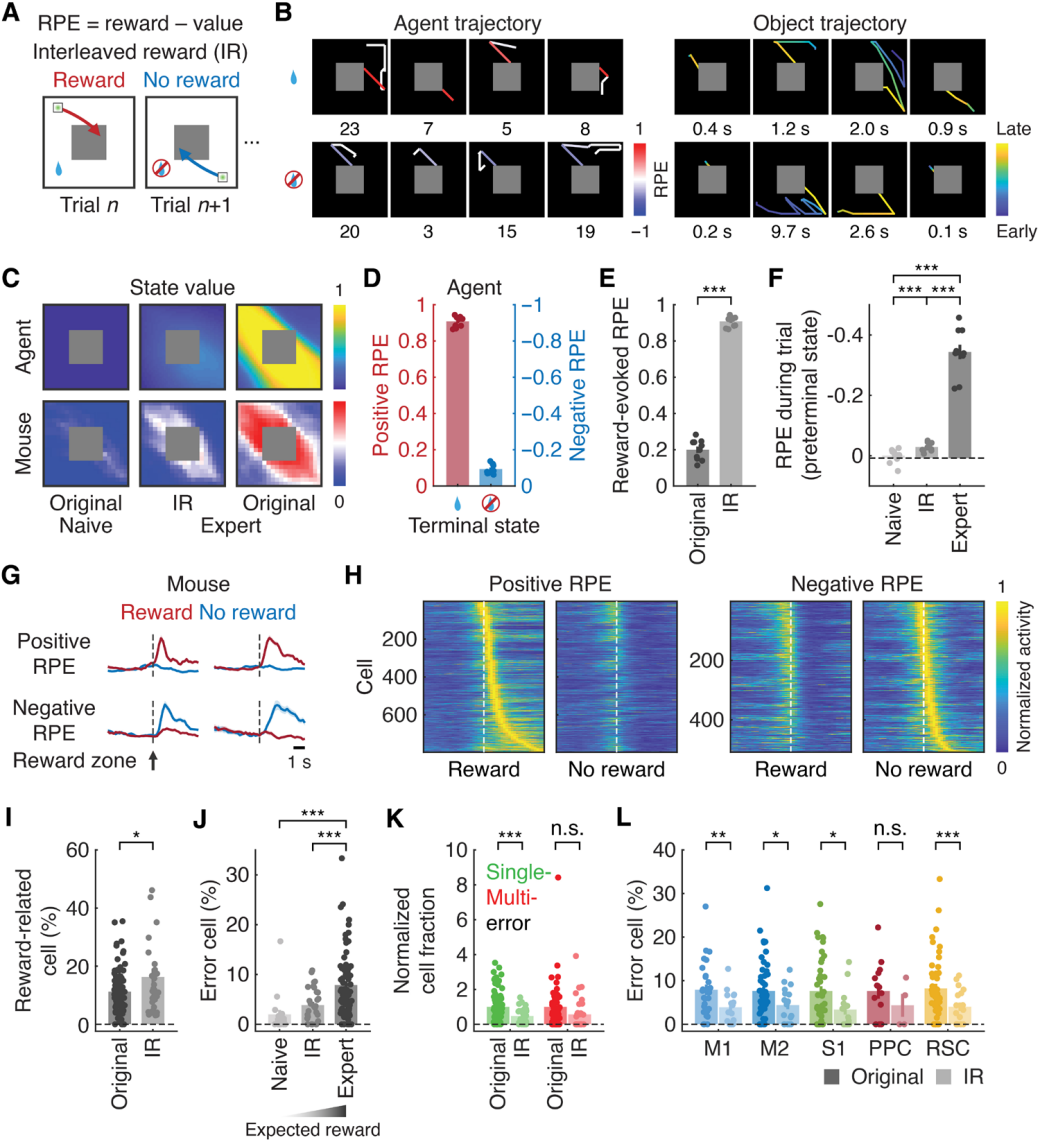
Reward dependency of error representations. (**A**) Schematic of the IR environment. (**B**) Example object trajectories in rewarded and nonrewarded trials in an expert agent (step number) and mouse (trial duration) in the IR environment. (**C**) Examples of critic output across different conditions. (**D**) Positive and negative RPE in expert agents in response to the presence or absence of a reward, respectively (*n* = 10 agents). (**E**) Reward-evoked RPE in expert agents (***P < 0.001, *n* = 10 agents, one-tailed bootstrap, means ± SEM). (**F**) RPE before reaching the reward zone (IR: expert) (***P < 0.001, *n* = 10 agents, one-tailed bootstrap with Bonferroni correction, means ± SEM). (**G**) Responses of example neurons encoding the positive RPE or negative RPE (means ± SEM). (**H**) Activity of classified neurons sorted by peak activity timing (positive RPE: 797 neurons; negative RPE: 509 neurons from five mice). (**I**) Fractions of reward-related neurons in expert mice (*P < 0.05, original: *n* = 75 sessions from nine mice; IR: *n* = 29 sessions from five mice, one-tailed bootstrap, means ± SEM). (**J**) Fractions of error neurons (IR: expert) (***P < 0.001, naive and expert: same as Fig. 3E; IR: *n* = 29 sessions from five mice, one-tailed bootstrap with Bonferroni correction, means ± SEM). (**K**) Fractions of single-step and multistep error neurons in expert mice normalized to original fractions (single-step: ***P < 0.001; multistep: n.s., *P* = 0.06, expert: same as Fig. 3F; IR: *n* = 29 sessions from five mice, one-tailed bootstrap, means ± SEM). (**L**) Fractions of error neurons in expert mice across cortical regions (*P < 0.05; **P < 0.01; ***P < 0.001; n.s., *P* = 0.15, expert: same as Fig. 3G; IR: M1: 16, M2: 18, S1: 15, PPC: 4, and RSC: 17 sessions from five mice, one-tailed bootstrap with Bonferroni correction, means ± SEM).

To study the nature of the observed error representations, we modified the task so that mice were rewarded in only half of the randomly interleaved trials [interleaved reward (IR) environment; Fig. 5A]. In this environment, we doubled the total number of trials to match the total reward available in the original environment. Mice were unable to predict, on a trial-by-trial basis, whether they would receive a reward after successfully moving the object to the reward zone. We predicted that the randomized reward delivery would allow us to examine both positive and negative RPEs upon the object reaching the reward zone. The positive RPE was defined as the difference between the actual and expected reward when the object reached the reward zone with a reward. The negative RPE corresponded to the same difference but occurred when a reward was omitted, although the object successfully moved to the reward zone (Fig. 5A). We hypothesized that the post–error activity of error neurons shared similar response properties with the activity reflecting the negative RPE defined here.

Theoretical analysis with deep RL verified the presence of both positive and negative RPEs when expert artificial agents reached the reward zone and either received or did not receive a reward, respectively (Fig. 5, B to D, and fig. S3C). As expected, the critic output in expert agents was smaller in the IR environment than in the original environment (Fig. 5C). Consequently, artificial agents exhibited higher reward-evoked positive RPEs in the IR environment than in the original environment (*P* < 0.001, *n* = 10 agents, one-tailed bootstrap; Fig. 5E). In addition, RPEs due to erroneous movements before reaching the reward zone were less negative in the IR environment compared to the original environment (*P* < 0.001, *n* = 10 agents, one-tailed bootstrap with Bonferroni correction; Fig. 5F).

In mice, the overall number of strokes and erroneous movements in the IR environment were comparable to those in the original environment (correct rate: 99.9 ± 0.06%, *n* = 29 sessions; trial-by-trial stroke number: 3.9 ± 0.1, *n* = 3480 trials from five mice, means ± SEM; Fig. 1, C and I, and fig. S1B). However, post–error anticipatory licking was reduced in the IR environment (*P* < 0.05, original: *n* = 75 sessions from nine mice; IR: *n* = 29 sessions from five mice, one-tailed Wilcoxon rank sum test with Bonferroni correction; fig. S1B), confirming the lower reward expectation.

To identify neural correlates of decision variables in mice, we analyzed deconvolved calcium signals and found neurons encoding positive or negative RPEs after the object reached the reward zone (Fig. 5, G and H). Consistent with the findings in artificial agents (Fig. 5E), the fraction of neurons responding to a reward was higher in the IR environment than in the original environment (*P* < 0.05, *n* = 75 sessions from nine mice and 29 sessions from five mice for the original and IR environments, respectively, one-tailed bootstrap; Fig. 5I). This suggests that these neurons did not simply respond to the reward itself.

We hypothesized that, if the error-related neural activity represented negative RPEs due to missing the reward zone, in the IR environment, fewer neurons would be activated following an erroneous movement, as the reward expectation was lower while the actual reward remained zero. In mice, after fitting GLMs (task-related cells: 60.7 ± 2.2%, *n* = 29 sessions from five mice, means ± SEM), we observed that the fraction of error neurons was lower in the IR environment than in the original environment (*P* < 0.001, original: *n* = 70 sessions from nine mice; IR: *n* = 29 sessions from five mice, one-tailed bootstrap with Bonferroni correction; Fig. 5J). A positive relationship emerged between the fraction of error neurons and reward expectation, as measured by anticipatory licking, across naive and expert mice in the original environment, as well as expert mice in the IR environment (*P* < 0.001, expert: 70 sessions from nine mice; naive: *n* = 19 sessions from five mice; IR: *n* = 29 sessions from five mice, one-tailed bootstrap for positive correlation; fig. S1B).

The reduction in the fraction of error neurons in the IR environment was observed for single-step errors, with a similar trend for multistep errors [single-step: *P* < 0.001; multistep: not significant (n.s.), *P* = 0.06, original: 70 sessions from nine mice; IR: *n* = 29 sessions from five mice, one-tailed bootstrap; Fig. 5K]. This reduction was consistent across all examined cortical areas except PPC (M1: *P* < 0.01, *n* = 27 and 16 sessions; M2: *P* < 0.05, *n* = 48 and 18 sessions; S1: *P* < 0.05, *n* = 44 and 15 sessions; PPC: n.s., *P* = 0.15, *n* = 14 and 4 sessions; and RSC: *P* < 0.001, *n* = 48 and 17 sessions from nine and five mice for original and IR, respectively, one-tailed bootstrap with Bonferroni correction; Fig. 5L). In addition, the area-specific code for n-step errors (Fig. 4, E and F) was replicated in the IR environment ($\delta^{\left(1\right)}$-$\delta^{\left(2\right)}$: n.s., *P* = 0.65, *R*^2^ = 0.06; $\delta^{\left(3\right)}$-$\delta^{\left(4\right)}$: *P < 0.05, *R*^2^ = 0.82; and $\delta^{\left(5\right)}$-$\delta^{\left(6\right)}$: *P < 0.05, *R*^2^ = 0.76, Pearson correlation coefficient computed with Student’s t cumulative distribution function, one-tailed; fig. S8, A to C).

Importantly, error neurons identified by the GLM before the object reached the reward zone and neurons encoding negative RPEs in response to the lack of a reward at the trial offset in the IR environment shared similar response properties. For example, the activity of both single-step and multistep error neurons was modulated by reward omission at the trial offset (fig. S9A). Furthermore, the activity of negative RPE neurons defined at the trial offset in the IR environment was greater than that of positive RPE neurons following each n-step erroneous movement (fig. S9B). Together, these results further support our view that the observed activation of error neurons depended on the lack of expected reward following erroneous movements.

### State value dependency of error representations

**Fig. 6.**
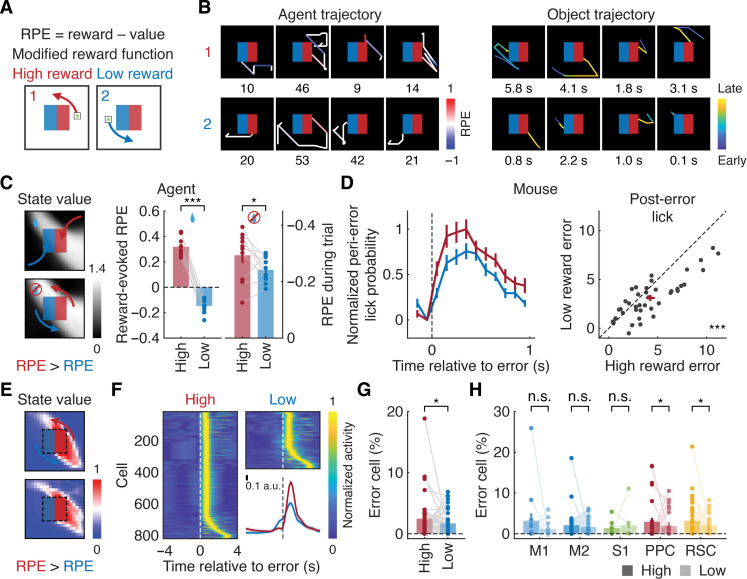
Value dependency of error representations. (**A**) Schematic illustrating RPEs in the environment with the modified reward function. (**B**) Example object trajectories in an expert agent (step number) and mouse (trial duration) in the environment with the modified reward function. (**C**) (Left) Examples of critic output representing state value in expert agents. Note the diagonal spread of high-value states caused by agents’ movement bias mimicking mouse behavior. (Right) Reward-evoked RPE (***P < 0.001, *n* = 10 agents, one-tailed bootstrap, means ± SEM) and RPE before reaching the reward zone in expert agents (*P < 0.05, *n* = 10 agents, one-tailed bootstrap, means ± SEM). (**D**) (Left) Normalized peri–error lick probability for errors related to failure to obtain a high or low reward. (Right) Session-by-session pairwise comparisons of post–error licking between the two types of errors (***P < 0.001, *n* = 41 sessions from six mice, one-tailed Wilcoxon signed-rank test, means ± SEM). (**E**) Examples of state value in expert mice. (**F**) Normalized GLM-derived activity of neurons responding to single-step errors for the two error types, sorted by peak activity timing (*n* = 805 and 362 neurons from six mice for high and low reward, respectively). (Inset) Mean GLM-derived activity of neurons encoding either of the two error types (*n* = 1167 neurons, means ± SEM). a.u., arbitrary units. (**G**) Fractions of error neurons for the two error types (*P < 0.05, *n* = 41 sessions from six mice, one-tailed paired bootstrap, means ± SEM). (**H**) Fractions of error neurons in each cortical region (M1: *P* = 0.20, *n* = 14 sessions; M2: *P* = 0.06, *n* = 34 sessions; S1: *P* = 0.12, *n* = 8 sessions; PPC: *P < 0.05, *n* = 28 sessions; and RSC: *P < 0.05, *n* = 35 sessions, one-tailed paired bootstrap with Bonferroni correction, means ± SEM).

To further elucidate the neural representations of errors, we modified the reward function so that mice received a high reward (10 μl) when the object reached one side of the reward zone and a low reward (1 μl) when it reached the other side (Fig. 6A). We hypothesized that the error signal would depend on the estimated value of each state, with a higher state value associated with the high reward side of the arena. We reasoned that, if error neurons encoded negative RPEs, we should observe a higher fraction of neurons responding to erroneous movements when they resulted in failure to obtain the high reward.

Artificial agents developed asymmetrical state value representations between the left (low reward) and right (high reward) sides of the arena (fig. S3D). Consequently, reward-evoked RPEs were larger when the agents reached the higher reward side (*P* < 0.001, *n* = 10 agents, one-tailed paired bootstrap; Fig. 6, B and C, and fig. S10A). RPEs before reaching the reward zones were more negative when agents missed the higher reward (*P* < 0.05, *n* = 10 agents, one-tailed paired bootstrap; Fig. 6, B and C, and fig. S10A).

In expert mice, the correct rate (99.7 ± 0.1%, *n* = 41 sessions from six mice, means ± SEM), trial-by-trial stroke number (4.2 ± 0.1, *n* = 2460 trials from six mice, means ± SEM), trial-by-trial error frequency, and error latency in the environment with the modified reward function were similar to those in the original reward configuration (Fig. 1, C and I, and fig. S1C). Reward expectation was higher following erroneous movements on the higher reward side, as evidenced by more frequent post–error licking (*P* < 0.001, *n* = 41 sessions from six mice, one-tailed Wilcoxon signed-rank test; Fig. 6D).

We analyzed neural activity in mice using two types of single-step errors—errors corresponding to missing either the high or low reward zone—as predictors in the GLM (task-related cells: 52.3 ± 2.4%, *n* = 41 sessions from six mice, means ± SEM). Similar to the artificial agents, asymmetrical state values emerged over learning (Fig. 6E). In addition, post–reward activity was generally higher when the object reached the higher reward zone (M1: *P* < 0.001, *n* = 3980 neurons; M2: *P* < 0.001, *n* = 18,969 neurons; S1: *P* = 0.36, *n* = 1872 neurons; PPC: *P* < 0.01, *n* = 5338 neurons; and RSC: *P* = 0.49, *n* = 12,511 neurons from six mice one-tailed paired bootstrap with false discovery rate; fig. S10B).

Consistent with the theoretical analysis, more neurons were activated in response to missing the higher reward (*n* = 805 and 362 neurons from six mice for the high and low reward, respectively; Fig. 6F). Session-by-session analysis confirmed this result (*P* < 0.05, *n* = 41 sessions from six mice, one-tailed paired bootstrap for the median; Fig. 6G). Region-by-region analysis revealed that PPC and RSC were primarily contributing to this difference, with a similar trend observed in M2 (M1: *P* = 0.20, *n* = 14 sessions; M2: *P* = 0.06, *n* = 34 sessions; S1: *P* = 0.12, *n* = 8 sessions; PPC: *P* < 0.05, *n* = 28 sessions; and RSC: *P* < 0.05, *n* = 35 sessions, one-tailed paired bootstrap for the median with Bonferroni correction; Fig. 6H). Because the observed error-evoked neural signals depended on state values, these results indicate that they did not reflect movement errors per se but corresponded to negative RPEs due to the lack of an expected reward.

## DISCUSSION

Behavior is shaped by error signals. In our task, errors were common even in expert mice, primarily due to insufficient object movement to reach the reward zone or inaccurate lateral aiming. We first used deep RL to theoretically demonstrate that the accumulation of these errors over time could improve learning in artificial agents. We then experimentally explored whether mouse cortical neurons represented error accumulation and examined the nature of the error signal by modifying the reward function of the environment. Our results indicate that the mouse cortex encodes RPEs in a distributed manner, with neurons representing various degrees of their accumulation.

Crucially, the observed error signals were not attributable to other movement-related covariates, such as those associated with the movement termination or licking, as more neurons were activated after erroneous movements than at the offset of single-stroke successful trials. Supporting this view, neural responses were also modulated by changes in the reward function, a finding consistent with previous research in nonhuman primates (*32*). Moreover, by isolating activity related to the mere passage of time in the GLM, we excluded time as a potential explanation for the observed neural activity.

Dissociating different classes of errors is often challenging (*33*). Prevailing theories of motor learning in adaptation are primarily model-based, suggesting that sensory prediction errors drive motor adaption by updating a forward model to minimize the difference between predicted and observed sensory consequences (*34*). However, growing evidence indicates that motor learning is also influenced by motivational feedback, such as reward and punishment. In these cases, RPEs are computed by detecting the difference between expected and actual rewards (*35*, *36*), especially when sensory feedback is not fully observable (*37*). Our findings demonstrate that neural activity responding to errors was modulated by changes in the reward function. These results, however, do not entirely rule out the possibility that some of the observed neural activity reflected sensory prediction errors (*38*).

In the present study, we focused on the accumulation of negative RPEs. This is due to the observation that expert mice anticipated a reward after each stroke, a behavior externally observable through their licking. Investigating positive RPEs, which typically occur when the actual reward exceeds the expected reward, would require multiple rewarding states throughout the trial and a behavioral measure indicating that the mice are not expecting a reward. It is plausible that the accumulation of positive RPEs is equally important and could be further explored in different behavioral contexts. Using the IR environment, we demonstrated that a separate population of neurons encoded positive RPEs, revealing diverse RPE representations that are considered to improve learning (*15*, *20*, *21*, *39*, *40*).

Our results indicate that the mouse cortex exhibits varying levels of negative RPE accumulation. We present evidence for neural representations of mixed levels of RPE accumulation across distributed cortical areas. Experimentally, specific ablations of RPE neurons are not feasible. Although theoretical analysis with deep RL highlights the significance of negative RPE accumulation in learning, its precise functions in the brain remain unknown. The cortical representations of RPE accumulation could serve as an eligibility trace, assigning credit to relevant states and actions to update their value estimates (*17*). Alternatively, it could reflect a covariate resulting from similar RPE processing elsewhere. Although we have demonstrated strong alignments between theory and experiment across different task environments, it is unlikely that the exact same deep RL algorithm is realized in the brain. Future research is required to further dissect the precise role of the RPE accumulation signal and how it is routed to other cortical and subcortical structures, such as the basal ganglia (*41*).

Mechanistically, the representations of multistep RPEs might arise through several possible means. One possibility is that neurons encoding single-step RPEs are hierarchically connected to neurons encoding multistep RPEs. Alternatively, subthreshold activity may gradually ramp up, reaching a threshold for specific multistep RPEs by integrating synaptic inputs. Dissecting neural circuits followed by functional characterization of RPE neurons could provide a more detailed understanding of how multistep RPEs are represented in the brain.

Our finding that higher-order cortical regions, such as RSC, display RPE accumulation over long horizons aligns well with a recent study demonstrating the region specificity of persistent value signals (*42*). It is unlikely that such area-specific representations of error accumulation are directly related to the intrinsic timescale of each cortical region, as we separated the time component of neural activity in the GLM. Because value coding is closely related to RPEs, RSC might have local machinery to update value estimates by recruiting other interconnected regions such as the hippocampus and related cortical and subcortical structures (*43*–*45*). Moreover, experimental evidence supporting direct connections from RSC to M2 (*46*) is consistent with our observation that M2 is well positioned to encode long-term RPE accumulation. Future studies will reveal the contributions of RPE signals to motor control within neural circuits involving these higher-order cortical areas.

## MATERIALS AND METHODS

### Animals

All procedures were in accordance with the Institutional Animal Care and Use Committee at Nanyang Technological University (protocol number: A22031). Transgenic mice were obtained from the Jackson Laboratory (CaMKII-tTA: 007004; TRE-GcaMP6s: 024742). The mice were housed in standard cages under a reversed light cycle (12 hours:12 hours), and experiments were generally performed during the dark period. Both male and female hemizygous mice were used. Experimental data from our previous study were reanalyzed, and the details are fully described (*24*).

### Surgery

The surgical procedure was previously described (*24*). Briefly, adult mice (between 7 weeks and 4 months old) were anesthetized with 1 to 2% isoflurane and a craniotomy (~7 mm in diameter) was performed around the bregma. An imaging window, constructed from a small (~6 mm in diameter) glass plug (#2 thickness, Fisher Scientific, 12-540-B) attached to a larger (~8 mm in diameter) glass base (#1 thickness, Fisher Scientific, 12-545-D) using an ultraviolet-curing adhesive (Norland, NOA 61), was placed over the craniotomy. After applying 1.5% agarose (Sigma-Aldrich, A9793-50G) to the window, a custom-built titanium headplate was implanted with cyanoacrylate glue and secured with black dental acrylic (Lang Dental, 1520BLK or 1530BLK). Buprenorphine (0.05 to 0.1 mg/kg of body weight), Baytril (10 mg/kg of body weight), and dexamethasone (2 mg/kg of body weight) were administered subcutaneously. The mice were monitored until they recovered from anesthesia.

### Object manipulation task

The behavioral task was previously described (*24*). Briefly, water-restricted mice were trained to manipulate a custom-made joystick with their right forepaw to remotely move a 3D-printed cube with a 525-nm LED (Thorlabs, LED525E) attached. The center of the reward zone (4 by 4 cm^2^) in the arena (10 by 10 cm^2^) was indicated by another 525-nm LED (Thorlabs, LED525E). Licking events were detected by a custom-made touch sensor. The task structure was controlled by Bpod (Sanworks), and the task variables were recorded by Wavesurfer (Janelia Research Campus) at a sampling rate of 2000 Hz.

Each session consisted of up to 60 trials (120 trials for the IR environment) over ~1 hour. The trial began with LED onset. When the object reached the reward zone, it was made stationary, and the LEDs were turned off. This was followed by a reward consumption period (4 s) and an intertrial interval (ITI; 2 s). After each successful trial, the object was reinitialized to a random position outside the reward zone. Each trial lasted up to 5 min, and if the object failed to reach the reward zone, then the trial ended with an ITI.

In the experiment with the modified reward function, when the object reached one side of the reward zone (right or bottom side), the mice received 10 μl of water. If it reached the other side (left or top side), then they received only 1 μl of water.

### Two-photon calcium imaging

Two-photon calcium imaging was described previously (*24*). Briefly, calcium imaging at layer (L) 2/3 of the cortex (~200 to 300 μm deep) was performed with a 2p-RAM (Thorlabs) with ScanImage (MBF Bioscience). The laser (InSight X3, Spectra-Physics) was tuned to 940 nm with the power of ~40 mW at the objective lens. The imaging was conducted at a frame rate of ~5.67 Hz, with a resolution of 1 × 0.4 pixel/μm, capturing two fields of view each measuring 0.5 mm × 5 mm. Imaging frames were recorded with Wavesurfer and aligned to behavioral events offline.

### Behavior data analysis

#### Correct rate

The correct rate was calculated by dividing the number of completed trials, in which the object was successfully guided to the reward zone, by the total number of trials. For the IR environment, the rate was calculated regardless of whether a reward was delivered. In the environment with the modified reward function, it was calculated regardless of the reward magnitude. The maximum number of trials was 60 for naive and expert mice in the original environment and the environment with the modified reward function and 120 for the IR environment.

#### Object movement and error

The object trajectory was subsampled every 10 ms. In each trial, a stroke was defined as an object movement with a speed exceeding 60 mm/s, separated by at least 200 ms from any previous movement. To compute an error, the time series of object movements approaching or moving away from (but not reaching) the reward zone were isolated for each trial. Concretely, the object’s distance to the center of the arena was calculated at each time point, and the differences between adjacent time points were obtained. The peak timing of the resulting time series was identified using MATLAB’s “findpeaks” function, applying a threshold of 60 mm/s and a minimal interval of 200 ms between errors. The time series of single-step errors were represented as a boxcar function, where a value of one was assigned when a single-step errors was detected and zero otherwise. For each value of n (n = 1, 2, 3, 4, 5, and 6), error accumulation, $\delta_{t}^{\left(n\right)}$, was represented in each trial as a boxcar function, with a value of one assigned when the cumulative error reached n and zero elsewhere. The error accumulation was reset to zero during the ITI. Trials shorter than 200 ms were not considered.

#### Peri–error lick

For each session, lick event time series were subsampled every 10 ms, and a peri–error lick histogram was generated for each n-step error. The data were binned every 100 ms by averaging, and the baseline (−50 ms relative to the error) was subtracted. These values were then normalized as follows: (i) to the maximum of the single-step peri-lick histogram of expert mice (object moving “toward”) for comparison between the “toward” and “away” conditions; (ii) to the maximum of the single-step peri-lick histogram of expert mice for comparison across expert, naive, and IR conditions; (iii) to the maximum of the single-step peri-lick histogram of the one-step error for comparisons across n within each trial; (iv) to the maximum of the single-step peri-lick histogram in the first quarter of each session for comparisons across trials; or (v) to the maximum of the single-step peri-lick histogram of the high reward side in the environment with the modified reward function. Lick frequency across conditions was compared by summing the first five-time bins following the error in the normalized histogram.

#### State value

The state value was previously defined as the mean discounted time steps for each spatial bin (*24*). Because the mice did not receive rewards outside of the reward zone, the state value was computed as

$$\text{State value}=E\left[\gamma^{T-t}\right]$$ (1)

where E represents the expectation, t is the time point taken every 10 ms, γ is the discount factor, 0.99, and T is the trial end when the mouse receives a reward of 1.

For the environment with the modified reward function, the state value was calculated in the same manner, except that the reward size was adjusted according to the amount of water given to the mouse relative to the original task (1.25 and 0.125 for the high and low reward zones, respectively).

### Imaging data analysis

#### Preprocessing

The imaging data preprocessing was previously described (*24*). Deconvolved calcium traces from cell bodies were obtained using Suite2p (https://github.com/cortex-lab/Suite2P) for image registration, semiautomated cell detection, and neuropil correction (*47*). Only neurons with activity surpassed a threshold of 20 at least once were included in further analysis.

Parcellation of the cortical areas was based on the Allen Mouse Common Coordinate Framework. Neurons were categorized into one of five cortical regions base on their distance from the bregma, which was located at the center of the imaging window. The number of analyzed cells in each region was as follows: naive: M1: 1077; M2: 1761; S1: 797; PPC: 207; and RSC: 1098; expert: M1: 7377; M2: 6215; S1: 7506; PPC: 1055; and RSC: 8625; IR experiment: M1: 2615; M2: 4019; S1: 1227; PPC: 133; and RSC: 3018; and experiment with the modified reward function: M1: 3980; M2: 18,969; S1: 1872; PPC: 5338; and RSC: 12,511. The number of task-related cells in each region was as follows: naive: M1: 789; M2: 954; S1: 608; PPC: 122; and RSC: 528; expert: M1: 4725; M2: 2935; S1: 4777; PPC: 520; and RSC: 2845; IR experiment: M1: 1870; M2: 2582; S1: 926; PPC: 73; and RSC: 1368; and experiment with the modified reward function: M1: 2538; M2: 10,766; S1: 1238; PPC: 3463; and RSC: 5228. Neurons located at the borders of cortical areas were not classified.

#### Generalized linear model

The encoding properties of experimentally designed task variables were independently modeled for each neuron using a GLM, as previously described (*24*, *29*). Task variables included n-step errors, object velocity, joystick velocity, and the passage of time for each trial. Because task variables were measured at a higher temporal sampling rate (2000 Hz) than the imaging (5.67 Hz), they were downsampled to match the imaging sampling rate.

The design matrix for the GLM was obtained as follows. The n-step (n = 1, 2, 3, 4, 5, and 6) error accumulation was represented as a boxcar function for each n, assigning a value of one at the onset and zero elsewhere. The angles of object velocity and joystick velocity were discretized into eight equally spaced bins (0°, 45°, 90°, 135°, 180°, 225°, 270°, and 315°), generating eight time series data with the amplitude of movement. The passage of time was a monotonic function corresponding to the number of frames within each trial. Each task variable was convolved with behaviorally appropriate temporal basis functions to produce task predictors. Six evenly spaced raised cosine functions, extended 2 s forward and backward in time, were used. In total, 138 task predictors (n-step error accumulation: 36; object velocity: 48; joystick velocity: 48; and passage of time: 6) were used to predict the deconvolved calcium signal for each neuron. As a control, a separate GLM design matrix was created by replacing the n-step error predictors with those derived similarly but using object movements away from the reward zone. The control design matrix also consisted of 138 task predictors (n-step movement accumulation: 36; object velocity: 48; joystick velocity: 48; and passage of time: 6).

For the environment with the modified reward function, only single-step errors were considered. The GLM design matrix was modified such that the original error predictors were replaced by two types of single-step error predictors corresponding to errors near the high or low reward zone. A total of 114 task predictors (two types of single-step error: 12; object velocity: 48; joystick velocity: 48; and passage of time: 6) were used to predict the deconvolved calcium signal for each neuron. Separate GLMs were fitted using control predictors corresponding to object movements away from the reward zone. This control matrix included 108 task predictors (single-step movement: 6; object velocity: 48; joystick velocity: 48; and passage of time: 6).

#### GLM fitting

The GLM fitting procedure was previously described (*24*). Briefly, task predictors were *z*-scored across the entire session, and the data were divided into a training set (70% of image frames) and a test set (30% of image frames) for each session. GLMs were fitted to each neuron’s activity using the “lassoglm” function in MATLAB with fivefold cross-validation on the training data. Elastic net regularization was applied with a ratio of α set to 0.9 (0.9 lasso regularization and 0.1 ridge regularization). Model performance was assessed using the pseudo-E.V. on the test dataset, calculated as

$$\text{Pseudo}\;E.V.=1-\frac{D\left(\hat{y}\right)}{D\left(\bar{y}\right)}$$ (2)

where

$$D\left(\hat{y}\right)=\text{logL}\left(y\right)-\text{logL}\left(\hat{y}\right)$$ (3)

is the deviance from the saturated model in terms of log likelihoods, and

$$D\left(\bar{y}\right)=\text{logL}\left(y\right)-\text{logL}\left(\bar{y}\right)$$ (4)

is the deviance from the null model (*30*). The null model was based on the mean activity.

Neurons were defined as task-related cells if they exhibited a positive pseudo-E.V. that was statistically higher (*P* < 0.05) than that obtained by shuffling the task predictors 1000 times with 2-s bins and if at least one task variable contributed to their activity. For a task variable to be classified as contributing to the activity of a given neuron, its pseudo-E.V. had to be positive. In addition, each task variable was individually removed, and the reduction in overall pseudo-E.V. was statistically assessed (*P* < 0.05) by shuffling the task predictors 1000 times with 2-s bins.

The GLM estimates neural activity by exponentiating the weighted sum of the task predictors. Therefore, the estimated neural activity can be expressed as a product of the exponentiated task variables ($e^{a+b}=e^{a}\times e^{b}$) and can be decomposed into the activity contributions of each task variable (*31*). The model-defined response profile for a given variable was determined by marginalizing out the other variables.

#### Analysis of error representations

To exclude potential confounding variables, neurons were identified as error encoding when the coefficients of the error terms in the GLM were higher than those for the object velocity and joystick velocity terms. In addition, object movement away from the reward zone (reinitialization) was convolved with the same basis functions, and the original error predictors were replaced with these control predictors to fit separate GLMs. Neurons were considered error encoding if the coefficients for the errors were higher than those obtained for the control predictors. Only excitatory modulation in the model-derived response for each n-step error was considered. Because of these strict criteria aimed at avoiding false positives, the resulting number of error neurons is likely an underestimate.

To classify the error neurons for different n, the argmax of the coefficients across n was computed. In each cortical region, the fractions of task-related cells among analyzed cells and the fractions of error neurons among task-related cells were determined when there were more than five analyzed and task-related cells, respectively.

To further eliminate other covariates in the error neurons, such as movement termination, reward, and lick, responses to the single-step error and the trial offset in single-stroke successful trials were compared in neurons deemed to represent the single-step error. If the response was larger upon the single-step error than the response following the trial offset, then it was considered to be evoked by the error.

To determine whether a population of n-step error neurons was activated sequentially, the activity onset for each neuron in a given trial was identified as the time when the *z*-scored activity, derived from the entire session, exceeded a threshold of 2. For display purposes, because of the abundance of single-step error neurons, only those with a pseudo-E.V. greater than 0.2 were shown to highlight the neurons encoding multistep errors. Neurons were then sorted based on the order of activity onset in one half of the trials within each session (training set), and the sorting index was applied to the other half of the trials (test set). Linear regression was performed between the sorting index and the trial-averaged rank of these neurons in the test set to compute a slope. A slope of 1 indicates consistent sequential activity across trials, whereas a slope of 0 means no consistency. To determine whether the observed slope was not obtained by chance, the activity onset was shuffled 1000 times across neurons in each trial, and slopes from the resulting shuffled activity onset were computed. The slopes were averaged across sessions to compute animal-specific values and across mice to compute a population value.

To test whether the activity of the identified error neurons was evoked by the passage of time, long trials were identified in expert mice as those above the mean trial duration across all trials from all mice. These trials were further split into two groups depending on whether they contained n-step errors. Trial-averaged deconvolved calcium signals were computed for each n-step error neuron in the two trial types, and their log (base of 10) values were compared.

#### *Area-specific representations for* n*-step error accumulation*

To determine the relative representations of different n-step errors in each cortical region, GLM coefficients for each n in error neurons prior to the argmax operation were calculated, along with the fractions of n-step error neurons among all error-encoding neurons. For the GLM coefficients, *P* values for region-by-region comparisons were determined by shuffling sessions with replacement 1000 times. The fractions of n-step error neurons were normalized to the maximum across the five cortical regions for each n. To assess the statistical significance of the area-specific code, the fractions of n-step error neurons among task-related cells across all mice from the original and IR environments were computed for each region. Sessions were shuffled 1000 times with replacement, and the resulting numbers of n-step error neurons and task-related cells were combined. *P* values for region-by-region comparisons were then obtained using the 1000 shuffles.

To examine consistency of area-specific representations of for the n-step errors across the original and IR environments, error neurons were combined across sessions and animals for each n-step bins (1-2, 3-4, and 4-6). For each bin, the numbers were normalized so that the minimum and maximum for all regions in both environments were 0 and 1, respectively. Error bars represent the SD of normalized fractions of error neurons obtained by shuffling sessions.

#### Reward prediction error

In the IR experiment, peri–trial offset activity spanning 8 s was computed using the deconvolved calcium signal in both rewarded and nonrewarded trials. The trial offset was defined as the moment when the object successfully reached the reward zone, regardless of whether a reward was delivered. Activity was categorized into two classes: (i) positive RPE signal: This was identified when the mean activity level during the reward epoch (0 to 2 s relative to the trial offset) was higher than the baseline (−4 to −3.5 s relative to the trial offset), higher in rewarded than nonrewarded trials, and the maximum mean activity level during the post–trial offset epoch (0 to 2 s relative to the rewarded trial offset) was higher than during the pre–trial offset epoch (−2 to 0 s relative to the rewarded trial offset); and (ii) negative RPE signal: This was identified when the mean activity level during the epoch when the reward was expected but not delivered (0 to 2 s relative to the trial offset) was higher than the baseline (−4 to −3.5 s relative to the trial offset), higher in nonrewarded than rewarded trials, and the maximum mean activity level during the post–trial offset epoch (0 to 2 s relative to the nonrewarded trial offset) was higher than during the pre–trial offset epoch (−2 to 0 s relative to the nonrewarded trial offset). The significance of each condition was determined by a one-tailed bootstrap with a threshold of *P* < 0.01.

To investigate whether the reward-evoked neural activity in the IR experiment reflected the positive RPE signal, fractions of reward-responsive neurons were compared between the original and IR environments. Reward-responsive neurons were identified as those with a mean activity level during the reward epoch (0 to 2 s relative to the trial offset) higher than the baseline (−4 to −3.5 s relative to the trial offset). Significance was determined using a one-tailed bootstrap with a threshold of *P* < 0.001. The fractions of reward-responsive neurons were calculated on a session-by-session basis, and the differences between the original and IR environments were statistically evaluated with a one-tailed bootstrap.

#### Reward-evoked activity

To investigate learning-related changes in reward-evoked responses in the original environment, mean post–reward activity (0 to 0.5 s relative to reward delivery) of all analyzed neurons in both naive and expert mice was computed after subtracting the baseline activity measured at −4 s relative to reward delivery. The neurons were then categorized into different cortical regions.

A similar analysis was conducted to compare reward-evoked activity in response to high and low rewards in the environment with the modified reward function.

#### Comparisons of response properties of between error and RPE neurons

To determine whether the error neurons identified in the GLM share similar response properties with negative RPE neurons defined in the IR environment, peri–trial offset activity of single-step and multistep error neurons was compared between rewarded and nonrewarded trials. Conversely, the peri–error activity of neurons encoding positive and negative RPEs, as defined in the IR environment, was analyzed for each n-step error.

### Deep RL

#### Environment

The environment was constructed using Open AI’s gym framework, consisting of a continuous state space and a discrete action space. In this environment, an agent was tasked with navigating a two-dimensional space (−1.0 × 1.0 arbitrary units) from a random starting position to reach a reward zone located in the center of the arena (0.4 × 0.4 arbitrary units) (*24*). Upon successfully completing a trial, the agent received a reward of 1.0.

The state space comprised two variables: the x and y coordinates of the agent, ranging from −1.0 to 1.0 on both axes, confined within the environment‘s boundaries. The action space included 256 possible actions, with the first 192 (75%) corresponding to no movement and the remaining 64 actions (25%) representing movement in one of eight directions (0°, 45°, 90°, 135°, 180°, 225°, 270°, and 315°) at varying speeds. The speed had a diagonal bias, mimicking mouse behavior (minimum and maximum distance per step: 0°, 90°, 180°, and 270°: 0.216 and 0.720 arbitrary units; 45°, 225°: 0.072 and 0.240 arbitrary units; and 135°, 315°: 0.360 and 1.200 arbitrary units). Each trial concluded when the agent reached the reward zone or after the maximum number of steps per trial (60) was reached.

In the IR environment, a reward of 1.0 was provided in a randomly interleaved 50% of trials. In the environment with the modified reward function, the reward zone was spilt into two; the left reward zone provided a low reward (0.125), whereas the right reward zone offered a high reward (1.25).

#### Proximal policy optimization

A variant of PPO was implemented using PyTorch, with several modifications made to the standard PPO algorithm (*26*). The network architecture was a multilayer perceptron shared between the actor and critic, consisting of four hidden layers, each containing 256 units. The rectified linear unit was used as the activation function. The network took the state as input and produced the estimated state value and action probabilities using the softmax function.

To simulate the mouse experiment, single-step negative RPEs were considered in two scenarios: (i) when the agent did not move far enough to reach the reward zone and (ii) when it aimed for the reward zone too laterally. Because mice expected a reward after each stroke, single actions were assumed to be sufficient to reach the terminal state. Consequently, the single-step RPE was defined simply as the difference between the actual (or lack of) reward and the estimated value of the current state, without considering the discounted value of the next state, as in the canonical TD error. As in the case of the mouse experiment, the single-step RPE was disregarded when the agent moved away from the reward zone.

The agent was trained with a discount factor γ of 0.95 and n value of 1, 2, 4, 6, or 8 for the n-step RPE. After n steps before reaching the terminal state, the generalized advantage estimator (GAE; λ = 0.95) was computed for the PPO update. The GAE represents a mixture of n-step RPE accumulations, defined as (*18*)

$$\begin{array}{c} {\hat{A}}_{t}^{\left(1\right)}≔\delta_{t}\\ {\hat{A}}_{t}^{\left(2\right)}≔\delta_{t}+{\gamma \lambda \delta }_{t+1}\\ {\hat{A}}_{t}^{\left(3\right)}≔\delta_{t}+\left(\gamma \lambda \right)\delta_{t+1}+{\left(\gamma \lambda \right)}^{2}\delta_{t+2}\\ \ldots \\ {\hat{A}}_{t}^{\left(n\right)}≔\delta_{t}+\left(\gamma \lambda \right)\delta_{t+1}+\ldots +{\left(\gamma \lambda \right)}^{n-1}\delta_{t+n-1} \end{array}$$ (5)

The actor-critic networks were updated using the Adam optimizer with a learning rate of 1 × 10^−5^. The total loss was computed by combining the policy loss (coefficient: 1), value loss (coefficient: 0.2), and entropy for exploration (coefficient: 0.02) over 4000 episodes. Gradients were clipped (ϵ for PPO: 0.2; max norm: 0.4) to avoid large updates. The PPO update was repeated 10 times for each *n*-step iteration. During evaluation, stochastic policies were used to mimic the mouse behavior.

To ensure robustness in the results, a hyperparameter space was explored, varying the learning rate (1 × 10^−4^, 1 × 10^−5^, or 1 × 10^−6^), γ (0.95, 0.99, or 1), λ (0.95 or 1), value loss coefficient (0, 0.2, or 0.4), and entropy coefficient (0, 0.02, or 0.04).

#### Analysis of artificial agents

Naive agents were defined as those before the first episode, whereas expert agents were defined as those based on the average from the 3600th to the 4000th episodes. Positive RPEs were defined when the agent reached the reward zone and received a reward. Negative RPEs were defined when the agent reached the reward zone but failed to receive a reward in the IR environment. Reward-evoked RPEs were treated the same as positive RPEs, whereas RPEs during the trial before reaching the reward zone were determined by the difference between the actual reward and the estimated state value.

The state value was computed by inputting 100,000 randomly selected numbers ranging from −1.0 to 1.0 into the model. These state values were sorted into 40 × 40 bins and averaged within each bin. To study negative RPEs resulting from missing a high or low reward in the environment with the modified reward function, RPEs were categorized based on the current state (whether the agent was in the left or right half of the arena).

Unit tuning for spatial location in the last layer of the network was computed as the value representation. Unit tuning for action direction in the last layer of the network was computed by averaging the activity for each action direction based on the angle of the action.
