# Supplementary material for: Distributed representations of temporally accumulated reward prediction errors in the mouse cortex
Source: Sci Adv. 2025 Jan 22;11(4):eadi4782. doi: 10.1126/sciadv.adi4782 (PMC11753378; doi:10.1126/sciadv.adi4782)
Supplement: Supplementary file 1 — Figs. S1 to S10 [file sciadv.adi4782_sm.pdf]

Science Advances
AAAS

Supplementary Materials for

Distributed representations of temporally accumulated reward prediction errors in the mouse cortex

Hiroshi Makino and Ahmad Suhaimi

Corresponding author: Hiroshi Makino, hmakino@ntu.edu.sg

Sci. Adv. **11**, eadi4782 (2025)
DOI: 10.1126/sciadv.adi4782

This PDF file includes:

Figs. S1 to S10

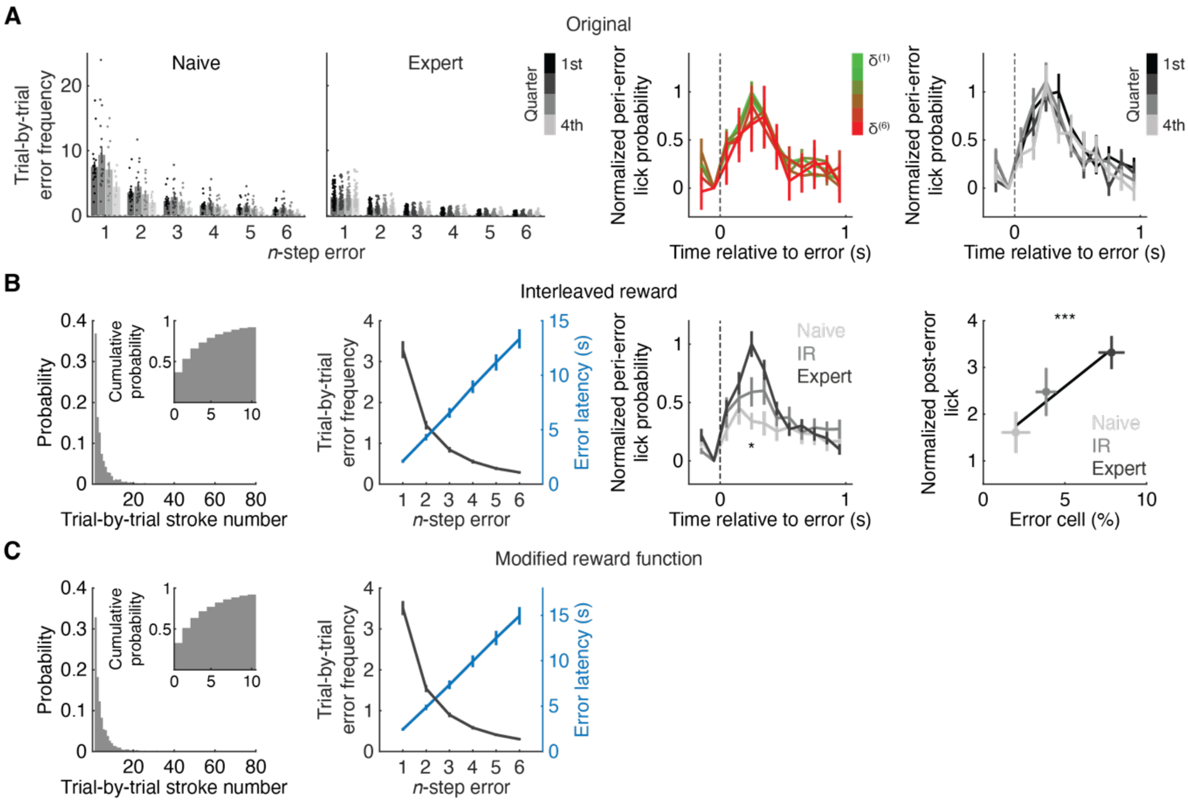

Figure S1. Additional analysis of behavior.

(A) Original environment. Left. Trial-by-trial error frequency across trials (every 15 trials) in naive and expert mice (naive: n.s., $P > 0.05$ for all n steps, n = 19, 18, 13, 8 sessions for the 1st, 2nd, 3rd, and 4th quarter from 5 mice; expert: n.s., $P > 0.05$ for all n steps, n = 75 sessions for all quarters from 9 mice, Kruskal-Wallis test with false discovery rate, mean ± SEM). Middle. Normalized peri-error lick probability across different n steps (n.s., P = 0.30, n = 75 sessions for 1-5 steps and 71 sessions for 6 steps from 9 mice, Kruskal-Wallis test, mean ± SEM). Right. Normalized peri-error lick probability across trials (every 15 trials, n.s., P = 0.60, n = 75 sessions for all quarters from 9 mice, Kruskal-Wallis test, mean ± SEM).

(B) IR environment. Left. Histogram and cumulative probability (inset) of trial-by-trial stroke number (n = 3480 trials from 5 mice). Middle left. Trial-by-trial error frequency and error latency as a function of n steps (n = 29 sessions from 5 mice for 1-6 steps, mean ± SEM). Middle right. Normalized peri-error lick probability in naive mice in the original environment and expert mice in the original and IR environments (*$P < 0.05$ between expert original and expert IR, expert and naive: same as **Figure 1G**, IR: n = 29 sessions from 5 mice, one-tailed Wilcoxon rank sum test with Bonferroni correction, mean ± SEM). Right. Positive relationship between fractions of error neurons derived from **Figure 5J** and normalized post-error lick probability (***$P < 0.001$, one-tailed bootstrap for positive correlation, mean ± SEM).

(C) Modified reward function environment. Left. Histogram and cumulative probability (inset) of trial-by-trial stroke number (n = 2460 trials from 6 mice). Right. Trial-by-trial error frequency and error latency as a function of n steps (n = 41 sessions from 6 mice for 1-6 steps, mean ± SEM).

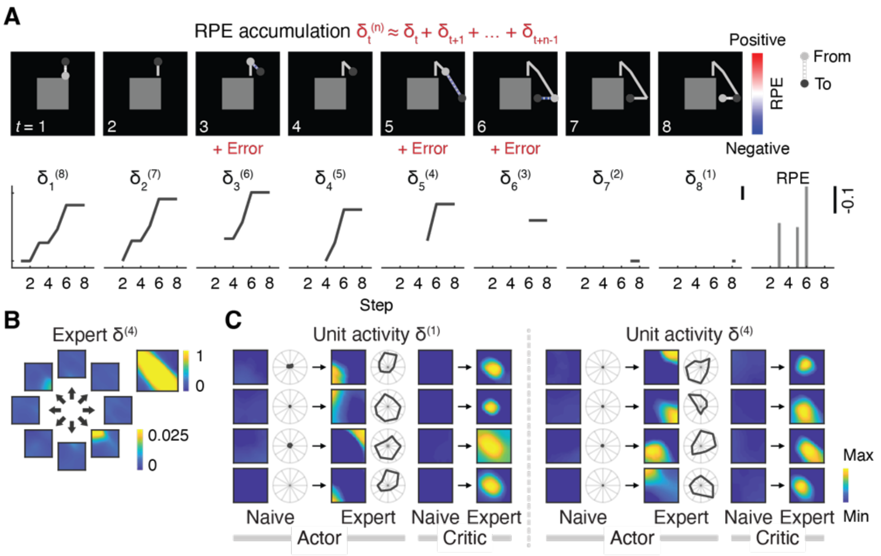

Figure S2. Accumulation of RPEs over time in RL.

(A) Example of RPE accumulation over time for different n steps.

(B) Example of actor and critic output from the expert neural network using $\delta^{(4)}$.

(C) Examples of the agent's unit representation in space and direction of actor and critic over learning with $\delta^{(1)}$ and $\delta^{(4)}$.

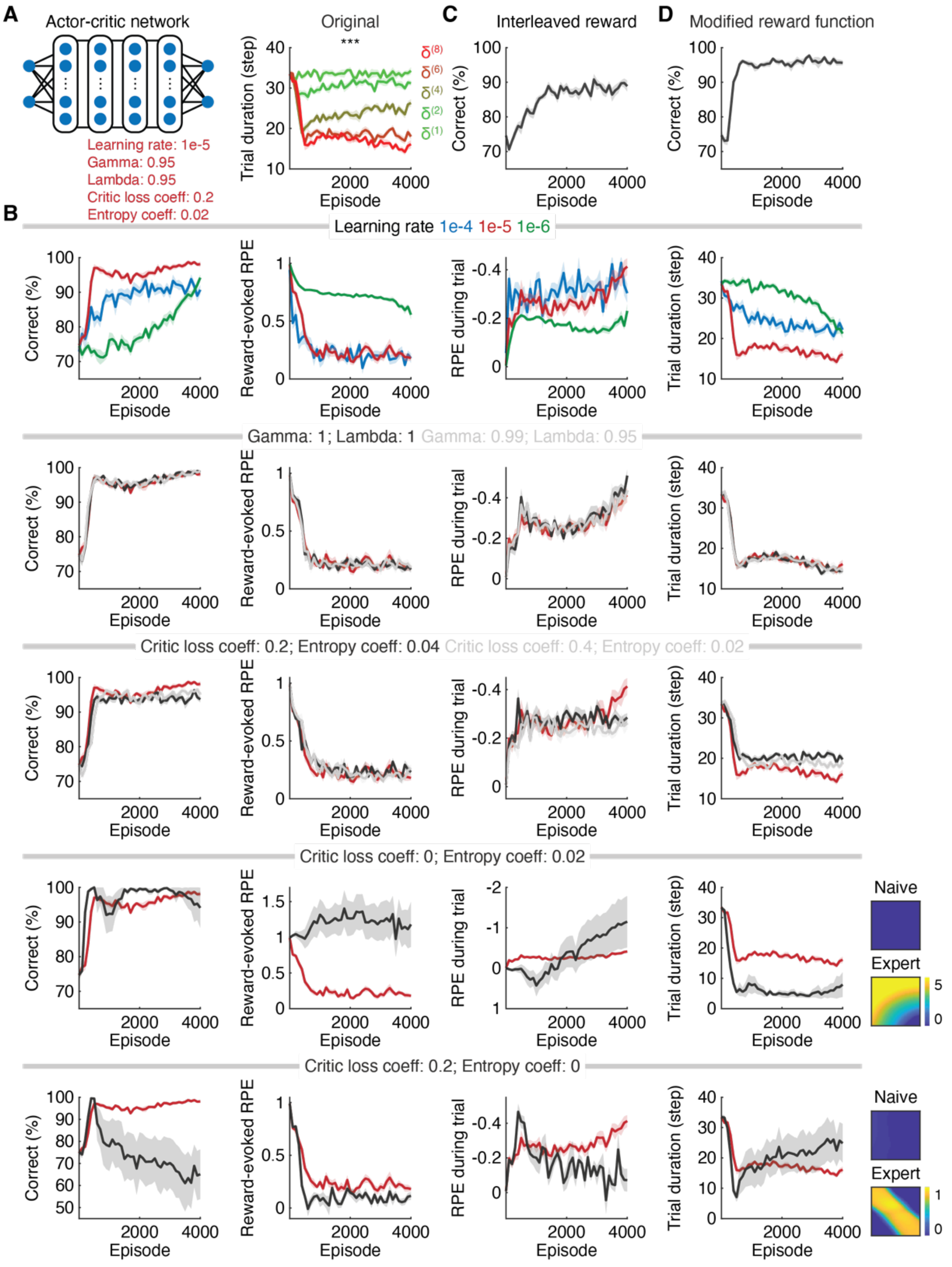

Figure S3. Additional analysis of artificial agents.

(A) Left. Actor-critic neural network and default PPO hyperparameters used to train artificial agents. Right. Learning-related changes in trial duration across different n steps (***P < 0.001, n = 10 agents, Kruskal-Wallis test, mean ± SEM).

(B) Left. Learning curves, learning-related changes in reward-evoked RPE, learning-related changes in RPE before reaching the reward zone, and learning-related changes in trial duration across different hyperparameters (n = 10 agents, mean ± SEM). The red lines were derived from the default hyperparameters. Right. Examples of critic output when the critic loss coefficient or entropy coefficient was set to 0.

(C) Learning curve for the IR environment (n = 10 agents, mean ± SEM).

(D) Learning curve for the environment with the modified reward function (n = 10 agents, mean ± SEM).

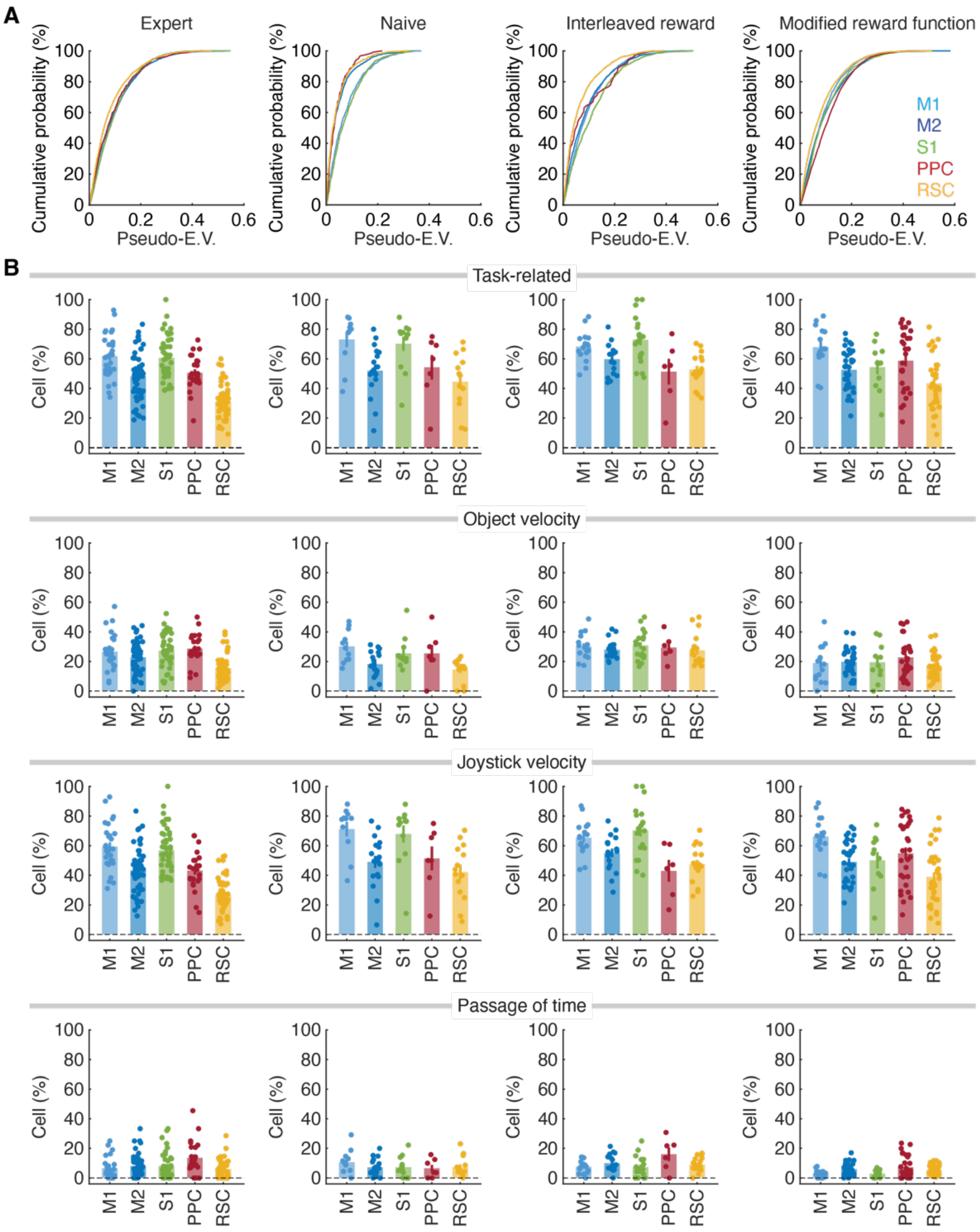

Figure S4. Pseudo-explained variance (E.V.) of task-related cells and their fractions.

(A) Pseudo-E.V. of task-related cells in each cortical region (expert: M1: 4725; M2: 2935; S1: 4777; PPC: 520; RSC: 2845 neurons from 9 mice; naive: M1: 789; M2: 954; S1: 608; PPC: 122; RSC: 528 neurons from 5 mice; IR: M1: 1870; M2: 2582; S1: 926; PPC: 73; RSC: 1368 neurons from 5 mice; modified reward function: M1: 2538; M2: 10766; S1: 1238; PPC: 3463; RSC: 5228 neurons from 6 mice).

(B) Fractions of task-related cells and cells encoding each task variable among analyzed cells in each cortical region across different experimental conditions (expert: M1: 28; M2: 55; S1: 44; PPC: 24; RSC: 55 sessions from 9 mice; naive: M1: 11; M2: 18; S1: 12; PPC: 7; RSC: 14 sessions from 5 mice; IR: M1: 16; M2: 18; S1: 19; PPC: 6; RSC: 17 sessions from 5 mice; modified reward function: M1: 15; M2: 35; S1: 11; PPC: 29; RSC: 35 sessions from 6 mice, mean $\pm$ SEM).

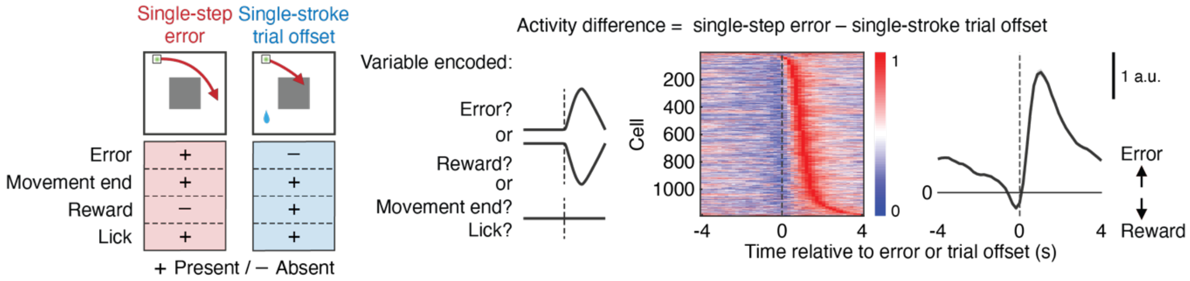

Figure S5. Comparison of neural responses encoding the single-step error between the single-step error onset and the trial offset of single-stroke successful trials in expert mice. Left. Schematic illustrating the comparison and hypotheses regarding the activity difference. "+" and "–" represent the presence and absence of the variable in each type of object movement, respectively. If neurons encode the single-step error, the activity difference should be positive. If neurons respond to the reward, the activity difference should be negative. If neurons encode the movement termination or licking behavior, the activity difference should be flat. Right. The neural activity difference between the two types of object movements (mean ± SEM). The difference is positive, indicating that these neurons encode the single-step error (or lack of expected reward), rather than the other variables. Of note, although unlikely, it is possible that their activation is triggered by the absence of a state transition indicator, such as the lack of LED offset.

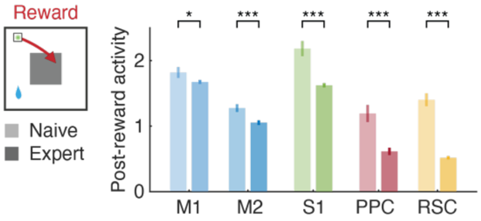

Figure S6. Changes in reward-evoked activity over learning.
Changes in post-reward activity across different cortical regions (*P < 0.05, ***P < 0.001, M1: n = 1077, 7619 neurons; M2: n = 1761, 6415 neurons; S1: n = 797, 7785 neurons; PPC: n = 207, 1081 neurons; RSC: n = 1098, 8776 neurons from 5 naive and 9 expert mice, one-tailed bootstrap with false discovery rate, mean ± SEM).

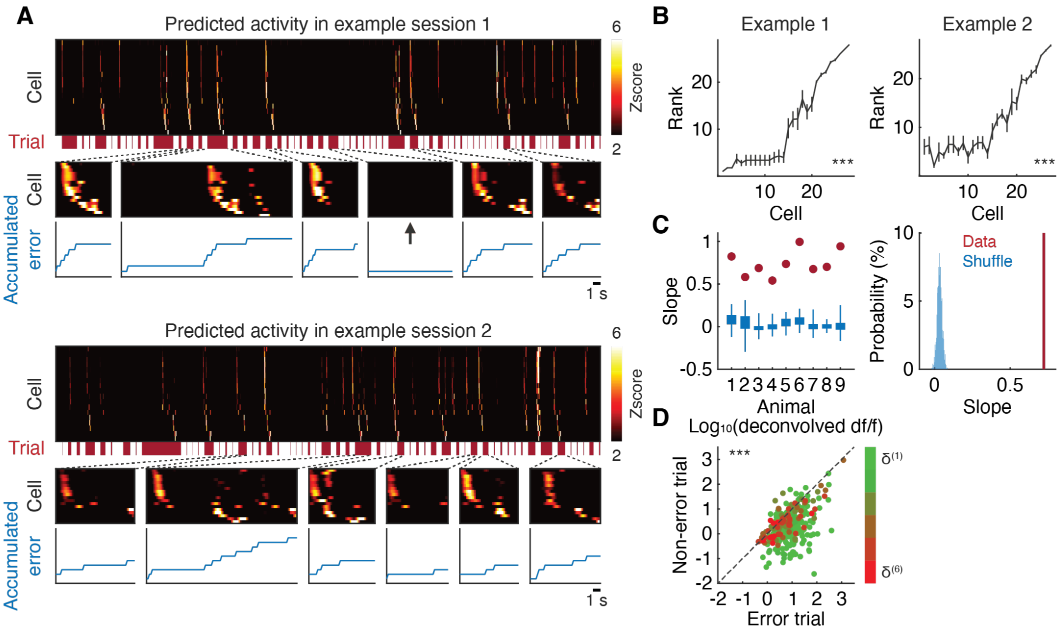

Figure S7. Sequential activity for error representations in expert mice.

(A) Example sessions displaying trial-by-trial sequential activity of cortical neurons. Top. z-scored GLM-derived activity of error neurons. Trial epochs are indicated by the red boxes. Middle. Example trials. The arrow highlights a long trial without errors, coinciding with the absence of neural activity. Bottom. Trial-by-trial error accumulation derived from the object trajectory.

(B) Mean ranks of activity onset of neurons across trials as a function of the sorting index from the example sessions in **(A)** (***P < 0.001, one-tailed permutation).

(C) Left. Sequential activity derived from GLM is not explained by chance in individual mice (P < 0.001 in all 9 mice, one-tailed permutation with Bonferroni correction). The whisker edges represent maximum and minimum values, and the box edges represent 75% and 25% of 1000 shuffled mean slopes. Right. Mean slope computed from all mice and mean slope derived from shuffled data of all mice (P < 0.001, n = 9 mice, one-tailed permutation).

(D) Higher mean activity derived from the deconvolved df/f of the GCaMP6s signal in n-step error neurons during erroneous trials compared to non-erroneous trials across different n steps (***P < 0.001, n = 1333 cells from 9 mice, one-tailed bootstrap). Trials with durations longer than the mean trial duration across all trials from all expert mice were selected first, and then further split based on whether they contained n-step errors.

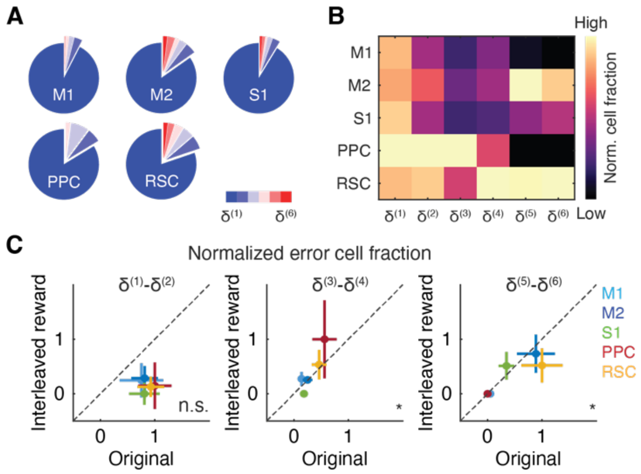

Figure S8. Area-specific code for error representations.

(A) Distribution of neurons encoding different n-step errors in each cortical region. Results from the original and IR environments were combined in panels **(A)** and **(B)**.

(B) Fractions of error neurons normalized to the maximum across regions in each n-step error.

(C) Consistency of the region-specific code for n-step errors across the original and IR environments ($\delta^{(1)}$-$\delta^{(2)}$: n.s., P = 0.65, R^2 = 0.06; $\delta^{(3)}$-$\delta^{(4)}$: *P < 0.05, R^2 = 0.82; $\delta^{(5)}$-$\delta^{(6)}$: *P < 0.05, R^2 = 0.76, Pearson correlation coefficient computed with Student's t cumulative distribution function, one-tailed, mean $\pm$ SD over 1000$\times$ shuffle). Fractions of error neurons were normalized across the two environments in each bin.

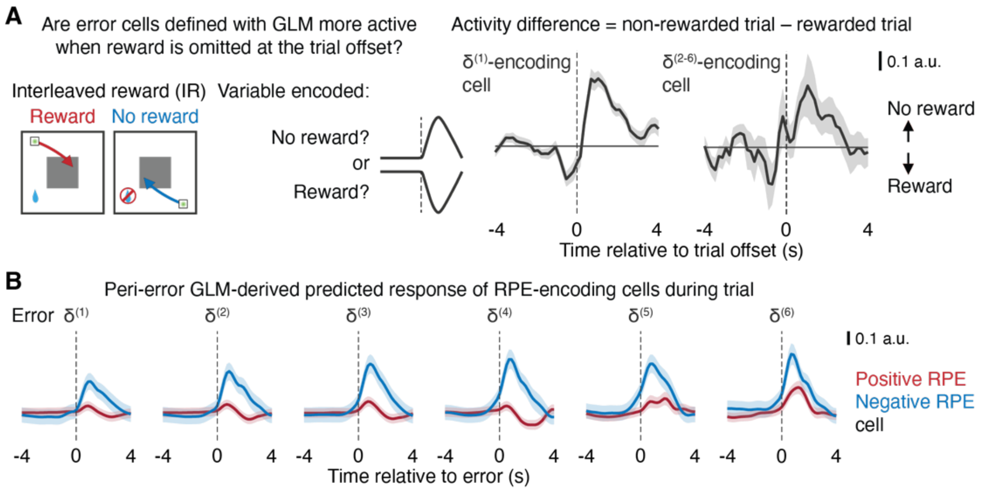

Figure S9. Error neurons, as defined by the GLM, share similar response properties with negative RPE neurons identified in the IR environment.

(A) Left. Schematic illustrating the comparison and hypotheses regarding the peri-trial offset activity difference between rewarded and non-rewarded trials. If error neurons, as defined by the GLM, respond to the absence of a reward, the activity difference should be positive. Conversely, if these neurons respond to the presence of a reward, the activity difference should be negative. Right. The neural activity difference between the two types of trials (mean $\pm$ SEM). The positive difference for n-steps 1 and 2-6, indicates that these neurons share similar response properties with negative RPE neurons.

(B) Peri-error GLM-derived predicted activity of positive and negative RPE neurons identified in the IR environment (**Figure 5H**), aligned to the error onset for different n steps. Negative RPE neurons defined in the IR environment exhibit similar response properties to error neurons.

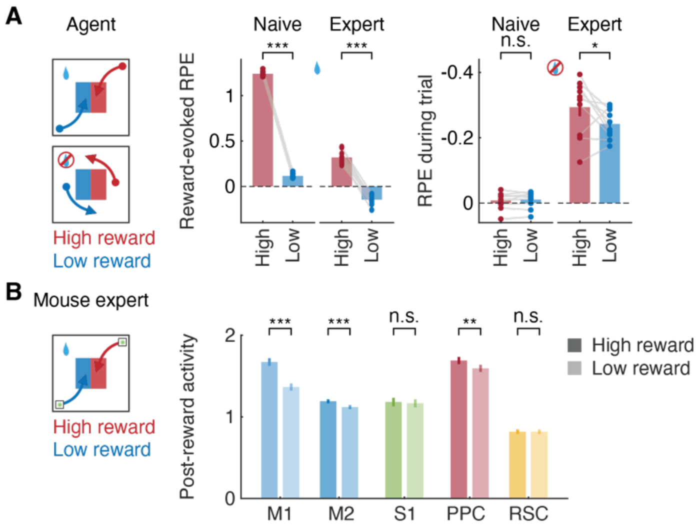

Figure S10. Additional analysis of the environment with the modified reward function.

(A) Left. Schematic of reward-evoked RPE and RPE before reaching the reward zone for high and low reward zones. Middle. Reward-evoked RPE over learning in artificial agents (naive: ***$P < 0.001$, n = 10 agents; expert: ***$P < 0.001$, n = 10 agents, one-tailed paired bootstrap, mean ± SEM). Right. RPE before reaching the reward zone over learning (naive: P = 0.76, n = 10 agents; expert: *$P < 0.05$, n = 10 agents, one-tailed paired bootstrap, mean ± SEM).

(B) Post-reward activity for high and low rewards in each cortical region (M1: ***$P < 0.001$, n = 3980 neurons; M2: ***$P < 0.001$, n = 18969 neurons; S1: P = 0.36, n = 1872 neurons; PPC: **$P < 0.01$, n = 5338 neurons; RSC: P = 0.49, n = 12511 neurons from 6 mice, one-tailed paired bootstrap with false discovery rate, mean ± SEM).
